# Compatibility of Insecticides with Rice Resistance to Planthoppers as Influenced by the Timing and Frequency of Applications

**DOI:** 10.3390/insects13020106

**Published:** 2022-01-18

**Authors:** Finbarr G. Horgan, Ainara Peñalver-Cruz

**Affiliations:** 1 EcoLaVerna Integral Restoration Ecology, Bridestown, Kildinan, T56 P499 County Cork, Ireland; 2 Centre for Pesticide Suicide Prevention, University/BHF Centre for Cardiovascular Science, University of Edinburgh, Edinburgh EH16 4TJ, UK; 3 Escuela de Agronomía, Facultad de Ciencias Agrarias y Forestales, Universidad Católica del Maule, Casilla 7-D, Curicó 3349001, Chile; 4 Institut de Génétique, Environnement et Protection des Plantes (IGEPP), Institut National de Recherche pour l’Agriculture, l’Alimentation et l’Environnement (INRAE), Institut Agro, Université de Rennes, CEDEX, 49045 Angers, France; ainara.penalver@agrocampus-ouest.fr; 5 International Rice Research Institute, Makati 1226, Metro Manila, Philippines

**Keywords:** *BPH32*, cypermethrin, deltamethrin, hormesis, phytotoxicity, prophylactic insecticides, resurgence, secondary outbreak

## Abstract

**Simple Summary:**

The brown planthopper, *Nilaparvata lugens* (Stål)(BPH) is a pest of rice in Asia. Varietal resistance is proposed as an alternative to insecticides that reduces BPH densities. However, in practice, resistance is often combined with insecticide use. We examined the effects of combining seven insecticides with resistance. We applied insecticides as one, two or three applications (experiment 1), or as early or late applications (experiment 2) to resistant (IR62) and susceptible (IR64) rice in a screenhouse environment. Carbofuran and fipronil reduced BPH biomass density. Single applications of cartap hydrochloride, cypermethrin, or buprofezin reduced BPH biomass densities on IR62, but not on IR64 (i.e., synergies); however, the effects were weak and multiple applications of all insecticides (≥2) eliminated synergies. Multiple applications of deltamethrin were antagonistic to resistance as indicated by higher densities of planthoppers on treated IR62 than on treated IR64. In non-infested plants from experiment 2, late applications reduced rice yields compared to early applications. Results suggest that early applications of some insecticides risk enhancing BPH densities, whereas late applications risk reducing rice yields. To avoid negative effects, applications should be made in compliance with Integrated Pest Management principals and multiple insecticide applications to BPH resistant rice should be avoided.

**Abstract:**

The brown planthopper, *Nilaparvata lugens* (Stål)(BPH) is a pest of rice in Asia. We examined the effects of seven insecticides combined with host resistance against BPH. In a screenhouse environment, we treated BPH-infested and non-infested resistant (IR62) and susceptible (IR64) rice with buprofezin, carbofuran, cartap hydrochloride, cypermethrin, deltamethrin, fipronil, or thiamethoxam + chlorantraniliprole. In one experiment, plants received one, two or three applications. In a second experiment, plants received one early or late insecticide application. Carbofuran and fipronil reduced planthopper biomass densities but resistance did not contribute to these effects (i.e., resistance was redundant). Single applications of cartap hydrochloride (at 20 or 50 days after sowing (DAS)), cypermethrin (20 DAS), or buprofezin (50 DAS) reduced BPH biomass densities on IR62 (i.e., synergies); other insecticides and application times, and multiple applications of all insecticides did not reduce BPH biomass densities on IR62 more than on IR64 (i.e., either resistance or insecticides were redundant). Deltamethrin (three applications) was antagonistic to resistance, but host resistance tended to buffer against the negative effects of single deltamethrin applications. Yields of infested IR62 were not statistically improved by insecticide applications. Late applications reduced yields of non-infested rice. We discuss how prophylactic insecticide applications could destabilize BPH populations and reduce the productivity and profitability of resistant rice.

## 1. Introduction

Rice is the principal staple food for over half of the world’s population [1]. About 90% of global rice production occurs in Asia and much of this is carried out in irrigated lowland environments [1,2]. Pressures to increase rice production in the Asian region have led to calls for crop intensification through the adoption of high-yielding varieties, increased mechanization, improved nutrient management and reductions to yield losses from pests and diseases [3]. Although several arthropod herbivores are capable of feeding on crop rice in Asia, only a few species have the potential to directly reduce rice yields at economically significant scales. These include a number of sap-sucking planthoppers and leafhoppers that cause direct mechanical damage to the rice plants, and/or transmit rice viruses that injure or kill the plants [4,5,6]. These sap-sucking insects are particularly difficult to manage because of their high reproductive capacity and, therefore, their ability to adapt to changing production environments, including changes to prominent rice varieties and agrochemicals [7]. Indeed, largescale outbreaks of planthoppers in particular, often follow regional changes to farming practices. For example, frequent outbreaks of non-migrating brown planthoppers, *Nilaparvata lugens* (Stål)(BPH), were first recorded in South and Southeast Asia at the time of the Green Revolution when farmers switched to high-yielding dwarf rice varieties, high fertilizer inputs and frequent insecticide use [4]. Furthermore, outbreaks of the white-backed planthopper, *Sogatella furcifera* (Horváth)(WBPH), became more prevalent following the widescale adoption of hybrid rice varieties and consequent changes to crop management practices [8].

Because planthopper outbreaks (BPH and WBPH) are associated with insecticide use [9,10,11], researchers have looked for alternative methods to manage these herbivores [3]. Over the last 50 years, public research has mainly focused on developing high-yielding rice varieties with resistance to planthoppers. Already, over 60 genes for resistance to planthoppers have been identified from traditional rice landraces or wild rice species [12]. Many of these have been incorporated into high-yielding varieties through marker assisted selection, and a number of varieties have been deployed to farmers’ fields [13,14]. However, intense research into host resistance has not resulted in any predictable decline in insecticide use. Indeed, during the time that research into host plant resistance has been most intense, insecticide use in Asia dramatically increased [3]. This implies that resistant rice varieties are likely to be treated with insecticides by farmers who are generally unaware of the resistance traits associated with their chosen varieties. This is significant because certain pesticides, and particularly pyrethroid insecticides, can increase the susceptibility of rice plants to herbivory by, for example, directly increasing sugar concentrations or reducing phenol concentrations [10,15,16,17]. Furthermore, planthoppers treated with pyrethroids sometimes exhibit enhanced fitness as part of a stress response to toxicity (i.e., hormesis) [17,18,19,20,21,22]. These effects are exacerbated where insecticide applications result in greater mortality of natural enemies than target herbivores, or where the predatory behaviors of natural enemies are impeded, thereby reducing the regulatory ecosystem services provided by, for example, parasitoids and spiders [7,9,19]. Therefore, insecticides could directly reduce the effectiveness of resistant rice varieties against planthoppers [7,20,23,24]. At field scales, such interactions between insecticides and crops of resistant rice can result in profitability losses for farmers [25] and erode the durability of resistance genes [26].

Integrated Pest Management (IPM) recommends that farmers only use insecticides when target pests exceed identified economic threshold densities [27,28]. However, ascribing threshold densities for different pests under variable climates and on varieties with different levels of resistance or tolerance is challenging, and published threshold densities can vary widely [29]. Furthermore, farmers are often unable to adequately monitor their rice fields to assess herbivore populations and the corresponding risks these herbivores pose to crop yields. In this context, agrochemical suppliers frequently promote prophylactic insecticide applications that are based on crop stages or calendar dates, and not on pest risks [30,31]. The potential for some pesticides to cause herbivore resurgence, combined with applications that are not correlated with risks, will destabilize planthopper populations. Resistant hosts can buffer against these effects; however, because target herbivores may never arrive to the crop, or never exceed threshold densities, and because certain pesticides will also directly reduce rice yields [24], prophylactic applications are more likely to lead to losses in rice profitability than are threshold-based applications.

In this study, we simulated rice production scenarios using potted plants in a screenhouse environment by introducing gravid BPH (representing dispersing populations) to rice plants at two management phases (i.e., before insecticide application, and after or between insecticide applications). We conducted the experiments in a screenhouse to exclude the effects of natural enemies in the study, because these represent an effective regulator of rice herbivore populations [7,8] and might therefore obscure the role of host resistance as a buffer against resurgence-causing insecticides. We conducted our experiments using the rice variety IR62 that is highly resistant to planthoppers in South and Southeast Asia [32,33], and compared this with an Asian mega-variety, IR64, that is susceptible to planthoppers [32,33]. We applied seven commonly used insecticides, without mixing products, during the experiments. We applied the insecticides at different frequencies (i.e., one, two or three applications) and at different growth stages (one application at 20 days after sowing or one at 50 days after sowing). We further assessed potential phytotoxic effects by applying each insecticide product to non-infested rice plants. Based on our understanding of the interactions between insecticides, planthoppers and rice, we identified a range of possible outcomes resulting from the application of host plant resistance and insecticides, either alone or in combination, on planthopper population development and ultimately on rice yields (Table 1). We predicted that the application of insecticides to resistant rice would function in synergy to reduce BPH population densities and increase yields [34]; however, we expected that repeated applications would reduce synergies because of increasing toxicity to the planthoppers. We also predicted that late applications of certain insecticides would have phytotoxic effects on rice plants that directly reduce grain filling [24]. We discuss our results in terms of the optimal management of resistant rice varieties in farmers’ fields.

## 2. Materials and Methods

### 2.1. Herbivore Species

BPH were obtained from colonies reared at the International Rice Research Institute (IRRI) in the Philippines. Planthoppers were initially collected in 2009 from rice fields in Laguna Province, Philippines. The founder population (ca 500 individuals) was placed in wire mesh cages of 120 × 60 × 60 cm (H × W × L) under greenhouse conditions (temperatures (T) = 25–37 °C; relative humidity (RH) = 70–90%, day: night (D:N) = 12 h:12 h) and was continuously reared on ≥30 days after sowing (DAS) TN1 rice plants. Feeding plants were replaced every two weeks. The colony received periodic introgressions of wild- caught individuals from the same collection sites. BPH from Laguna Province are adapted to feed on rice varieties with the *Bph1*, *bph2*, *bph5*, *bph7*, *Bph18*, *BPH25* and *BPH26* genes [32,33]. The colonies were also tested for levels of resistance to insecticides and had moderate resistance to imidacloprid and high resistance to fenobucarb (2-(Butan-2-yl)phenyl methylcarbamate; BPMC) [26].

### 2.2. Plant Materials

We used two varieties in our experiments. IR62 was selected as a variety with high resistance to BPH populations throughout South and Southeast Asia [35]. Resistance in IR62 is derived from the Indian landrace PTB33 and is attributed to the *Bph3* resistance locus that contains the *Bph3* and/or *Bph32* genes [35,36]. BPH has inefficient feeding, slower development and weight gain, and reduced fecundity and egg-laying on IR62 compared to susceptible varieties [37]. Resistance associated with the *Bph3* locus appears durable since BPH populations with virulence against IR62 remain rare despite > 30 years of deployment in some regions of the Philippines and Cambodia [38]. The variety IR64 has the *Bph1* gene for resistance, but BPH populations throughout Asia have already adapted to this gene [32,35] and the variety is therefore susceptible to BPH. IR64 is considered a mega-variety in Asia [39]. Because of its popularity, the variety has been enhanced using marker-assisted breeding to incorporate traits such as flood tolerance (i.e., IR64-Sub1, released in 2009) [39]. For our experiments, seeds of both varieties were acquired from the IRRI-germplasm bank.

### 2.3. Insecticides

We conducted our experiments with seven insecticides that are commonly applied to rice in Asia. The insecticides were selected based on their availability at IRRI. Buprofezin (25 SC; 200 mL ha^−1^) contains the growth regulator thiadezine and is used against planthoppers and leafhoppers in rice. We used two synthetic pyrethroids, cypermethrin (10 EC; 800 mL ha^−1^) and deltamethrin (25 EC; 500 mL ha^−1^): cypermethrin has been commonly used in rice production to combat rice tungro virus that is transmitted by rice leafhoppers [40]. Deltamethrin is a broad-spectrum insecticide. We used the carbamate insecticide carbofuran (3 G; 3 Kg ha^−1^) as a systemic and knockdown insecticide that is sometimes recommended for control of rice pests including planthoppers [41,42]. We used a thiamethoxam 20% + chlorantraniliprole 20% product (40 WG; 75 g ha^−1^) as a further systemic insecticide. Finally, we used fipronil and cartap hydrochloride (50 SP; 400 g ha^−1^) as two broad-spectrum insecticides commonly used to control rice leaf-folders in Asia [43,44]. Further details concerning these seven insecticides are presented by Horgan et al. (2021) [24]. All insecticides were applied at recommended rates (as indicated in parentheses) with quantities estimated based on the surface soil area of the potted rice. Insecticides were applied by trained pesticide applicators using standard protective equipment in a well-ventilated environment (an insect screenhouse). Any manipulations and evaluations of treated rice plants were conducted at a minimum of three days post-application.

### 2.4. Effects of Application Frequency on BPH and Host Plants

Seeds of IR62 and IR64 were initially sown in trays (one variety per tray) with saturated paddy soil. At 7 DAS, healthy seedlings were transplanted to pots of 22 × 24 cm (H × diameter—one plant per pot) in a screenhouse (T = 26–37 °C, RH = 70–90%, 12 h:12 h, D:N). Plants received ammonium (0.44 g), solophos (0.611 g) and muriate of potash (0.092 g) (basal application and at mid-tillering) equivalent to 100 kg ha^−1^. The plants were allowed to grow and develop for a further 10 days, after which, each pot was placed inside a mesh cage (130 × 24 cm (H × diameter)) supported by a stiff wire frame. The mesh was tied at the top and could be easily opened and closed to manipulate the plants.

At 20 DAS, plants (IR62 and IR64) were divided into seven groups (one group for each insecticide) of 84 plants each (2 varieties × 7 insecticides × 3 application frequencies × 2 infested/non-infested = 84; 84 × 5 replicates (for blocks) = 420 insecticide-treated pots). Each group of 84 pots was placed inside a plastic, open-top cubicle (3 × 2 × 1.5 m: L × W × H). The cages were opened at the top, and the plants were treated with one of the seven insecticides (each inside a separate designated plastic cubicle). After three days, the plants were moved out of the plastic cubicles and were repositioned into five randomized blocks. To each block, we added four pots with IR62 and four with IR64 plants (all pots were caged) as untreated infested and non-infested controls (i.e., (2 varieties × 7 insecticides × 3 application frequencies × 2 infested/non-infested + 2 non-treated, infested IR62 controls + 2 non-treated, non-infested IR62 controls + 2 non-treated, infested IR64 controls + 2 non-treated, non-infested IR64 controls) = 1 block of 92 plants × 5 replicated blocks = 460 pots in total). At 40 DAS, half of the plants in each block (3 pots with each variety × insecticide treatment + 4 controls) were infested with four gravid BPH females. The females were transferred from the greenhouse colony to the base of each plant using a suction pooter. At 50 DAS, two infested plants and two non-infested plants treated with each chemical were randomly taken from each block and again treated in designated cubicles with the corresponding insecticides as described above. After three days the plants were returned to their original blocks. The infested plants, including the infested non-treated controls, were again infested with two gravid female planthoppers at 60 DAS. Finally, at 80 DAS, one infested plant and one non-infested plant treated with two applications of each chemical were randomly taken from each block to the cubicles and treated with a third application of the corresponding insecticide as described above. After three days, the pots were returned to their original blocks. Each of the five blocks contained IR62 and IR64 plants under the following conditions: (1) insecticide at 20 DAS, BPH at 40 and 60 DAS; (2) insecticide at 20 and 50 DAS, BPH at 40 and 60 DAS; (3) insecticide at 20, 50 and 80 DAS, BPH at 40 and 60 DAS; (4) insecticide at 20 DAS; (5) insecticide at 20 and 50 DAS; (6) insecticide at 20, 50 and 80 DAS; (7) no insecticide, BPH at 40 and 60 DAS; and (8) no insecticide and no BPH. This sequence of infestations and treatments simulated prophylactic applications to infested and non-infested plants, with and without subsequent insecticide applications.

Plants were monitored daily. Planthoppers were collected from the plants when the plants appeared yellow (indicating the initiation of hopperburn) or when the grain was at >85% maturity. Plants that died at an early growth stage (i.e., before grain maturation) were noted. The planthoppers were collected from the infested plants using a battery-operated pooter (Hausherr’s Machine Works, Toms River, NJ, USA) and placed in plastic vials (one per pot). Planthoppers were killed by freezing, and were then placed inside paper envelopes. After the insects had been removed, the plants were allowed to further grow and develop. When plants had matured, as indicated by fully filled grain and final senescence, they were destructively harvested by carefully pulling each plant from the soil. Plants that died before maturation were harvested at about the time of death, with final plant biomass used to calculate BPH biomass density (see below). The roots were washed under running water and the plants separated into above-ground and below-ground portions. The plants were placed in paper bags and, together with the planthoppers, were dried in a forced draft oven at 60 °C until a constant weight. After drying, the planthoppers were counted and weighed. The plants were weighed and the numbers of tillers and panicles were recorded. Panicles were then removed and the grain separated as filled and non-filled grain. The grains were counted and weighed.

### 2.5. Effects of Application Timing on BPH and Host Plants

Plants of both varieties were planted to pots of 22 × 24 cm (H × diameter—one plant per pot) as described above. At 27 DAS, the plants were placed inside mesh cages (130 × 24 cm (H × diameter)) as described above. Plants were randomly positioned into five blocks of 64 plants (i.e., (2 varieties × 7 insecticides × 2 application times × 2 infested/non-infested + 2 non-treated, infested IR62 controls + 2 non-treated, non-infested IR62 controls + 2 non-treated, infested IR64 controls + 2 non-treated, non-infested IR64 controls) = 1 block × 5 replicated blocks = 320 plants in total).

At 20 DAS, 14 × IR62 and 14 × IR64 plants were randomly taken from each block and treated with one of the seven insecticides inside the plastic cubicles described above. After three days, the plants were returned to their corresponding blocks. At 40 DAS, half of the plants in each block (2 varieties × 7 insecticides × 2 application times + 2 non-treated IR62 controls + 2 non-treated IR64 controls) were infested with four gravid BPH females as described above. At 50 DAS, a further 14 × IR62 and 14 × IR64 untreated but infested plants were randomly taken from the blocks and treated with the insecticides as described above. The plants were returned to their corresponding blocks after three days. At 60 DAS, the infested plants in the blocks were again infested with two gravid BPH females. Each of the five blocks contained IR62 and IR64 plants under the following conditions: (1) insecticide at 20 DAS, BPH at 40 and 60 DAS; (2) insecticide at 50 DAS, BPH at 40 and 60 DAS; (3) insecticide at 20 DAS; (4) insecticide at 50 DAS; (5) no insecticide, BPH at 40 and 60 DAS and (6) no insecticide and no BPH. The experiment simulated early, prophylactic applications without infestation and at pre-infestation, and relatively late applications without infestation and at post-infestation.

The plants and insects were allowed to grow and develop until initial signs of hopperburn or until the grain was at >85% maturity at which time the planthoppers were collected. At final evaluation, the planthoppers and plants were collected and processed as described above.

### 2.6. Data Analyses

We divided both experiments into two parts. We first analyzed planthopper population parameters, as well as plant survival and anatomy for infested plants, and then assessed the possible phytotoxic effects of the pesticides on non-infested plants. Separating the analyses of infested and non-infested plants responded to separate hypotheses concerning synergies/antagonisms and phytotoxic effects, and to limitations on multi-factorial analyses with zero-infestation and non-treated controls, but with multiple levels of treated plants (see below). Because a large number of infested plants had died, or had reduced growth due to planthopper damage, we standardized densities and biomass per unit plant weight. We therefore present information on infestations in terms of the total numbers or biomass of insects per above-ground g of plant (henceforth density and biomass density, respectively). Because of the large numbers of plants used in the experiments, biomass density in particular is considered a convenient metric to assess relative planthopper fitness [45]. We present the results for BPH numbers per g of plant (density) only in the supplementary information.

We analyzed BPH population parameters (density, and biomass density) and plant growth parameters (tiller, panicle and grain numbers; shoot height and root length; above and below ground biomass; yields, 1000 grain weight and proportions of unfilled grain) using general linear models (GLM). We also analyzed the proportions of plants that had died under each treatment using GLM. Because our design included zero application controls (i.e., non-treated infested and non-infested controls) we used an extension of the Addelman [46] method for analyses of experiments with quantitative (i.e., application frequency = 1, 2 or 3; application time = 20 DAS or 50 DAS) and qualitative factors (i.e., insecticide active ingredients and controls) that include zero amounts. This allowed us to compare the relative effects of different insecticides against shared controls, where each insecticide was applied under two or more regimes, representing an additional factor that was not applicable to the controls. We further estimated the combined effects of host resistance and each insecticide ‘x’ as -((biomass density on treated IR62-biomass density on untreated IR64)/biomass density on untreated IR64). To estimate the effect of the insecticide alone on IR62 plants, we removed the effect of resistance which we estimated as ((biomass density on untreated IR62-biomass density on untreated IR64)/biomass density on untreated IR64) [24]. All calculations were based on plants within each block and using the average of each of the two controls for each block (i.e., non-treated, BPH-infested controls of IR62 or IR64). We then analyzed the combined and separate effects of insecticides using univariate GLMs. We initially included blocks as a random factor in all analyses; the factor was removed where it had no significant effect. Proportions were arcsine-transformed before analyses. We used Tukey pairwise comparisons to determine homogenous groups of pesticides or application frequencies after initial analyses. Residuals were plotted following all analyses and were normal and homogenous.

To further examine potential categories of outcome (Table 1), we also analyzed the effects of each insecticide on IR62 and IR64 using univariate GLMs where untreated controls were incorporated as a treatment type (i.e., treatments = 0, 1, 2 or 3 applications, or treatments = 0 applications, and applications at 20 or 50 days). This was possible because treatments were completely randomized within blocks for each experiment; these analyses did not differentiate qualitative differences between controls and treatments. The results of this second-level of analyses were, however, useful to explain significant interactions as identified using the primary Addelman-extended GLMs. Second level analyses were conducted for all insect parameters and for final yields with variety and application frequencies (experiment 1) or application times (experiment 2) as main factors. We used Dunnett’s many-to-one comparisons to test for significant application-related declines in BPH populations, or application-related increases in yields against non-treated controls. For experiment 1, we also tested for linear contrasts associated with application frequency. The main results of these second level analyses are presented in the main text, with full explanations included with the supplementary information. We included blocks as a random factor in all analyses and proportions were arcsine-transformed before analyses. Residuals were plotted following all analyses and were normal and homogenous.

## 3. Results

### 3.1. Effects of Host Resistance and the Frequency of Insecticide Applications on BPH Populations

**Figure 1:**
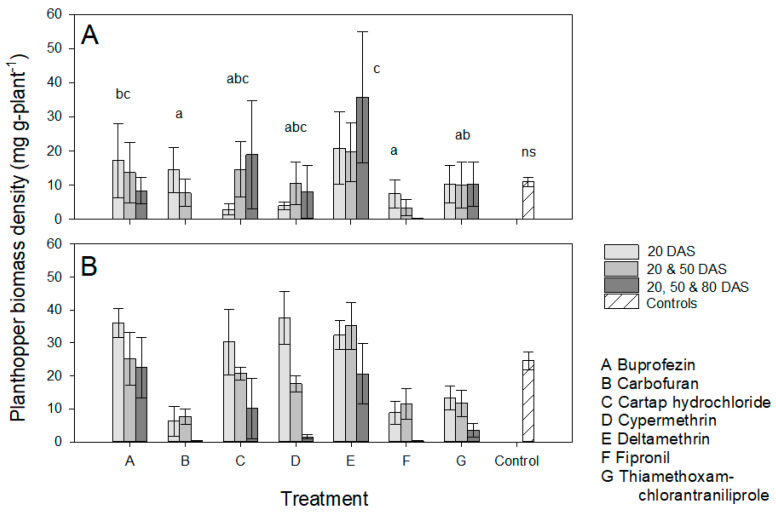
Planthopper biomass densities on (**A**) IR62 (resistant) and (**B**) IR64 (susceptible) rice plants treated with one (light gray), two (medium gray) or three (dark gray) applications of seven insecticides (x-axis, A–G). Non-treated, but infested control plants are indicated by hatched bars. All plants were infested with four gravid female BPH at 40 DAS and again with two gravid females at 60 DAS. Standard errors are presented (*n* ≤ 5 blocks). Lowercase letters indicate homogenous insecticide groups (i.e., within insecticide treatments) based on Tukey pairwise comparisons (*p* ≤ 0.05); ‘ns’ indicates no significant difference between insecticide treatments and non-treated, BPH-infested controls (*p* ≥ 0.05). Data related to this figure are also presented in Figure S1 with separate analyses for each insecticide presented in Table S2.

Planthoppers attained a higher biomass density (variety: F_1,170_ = 6.832, *p* = 0.010) (Figure 1) on IR64 plants, but there were no significant differences in planthopper densities on the two varieties (F_1,170_ = 0.611, *p* = 0.436: Table S1). The higher biomass density of planthoppers on IR64, resulted in higher proportions of plants dying due to herbivore damage (F_1,170_ = 11.443, *p* < 0.001: Table S1). There were no significant differences between infested controls, and treated plants in the proportions of plants that died (F_1,170_ = 0.043, *p* = 0.758: Table S1), in densities of BPH (F_1,170_ = 1.127, *p* = 0.289: Table S1), or in BPH biomass densities (F_1,170_ = 1.040, *p* = 0.309: Figure 1); variety × control interactions were also non-significant for each parameter (0.046 ≥ F_1,170_ ≤ 4.93, *p* < 0.05) (Table S1). Among treated plants, BPH biomass densities were lowest on carbofuran and fipronil-treated plants, and these were significantly lower than on buprofezin and deltamethrin-treated plants (F_6,170_ = 8.459, *p* < 0.001) (Figure 1). Carbofuran applied 2 or 3 times, and fipronil applied 1, 2 or 3 times reduced BPH biomass density below that of non-treated plants. Three applications of cypermethrin also reduced BPH biomass densities to below that of non-treated plants. Plants treated with thiamethoxam + chlorantraniliprole tended to have lower BPH biomass densities, but these were not significantly different from non-treated controls (Figure S1, Table S2). For carbofuran, cypermethrin and fipronil, significant variety × treatment interactions were due to lower biomass densities on non-treated IR62 plants, or on IR62 plants receiving a single application of cypermethrin (Figure S1). More carbofuran and fipronil-treated plants survived the infestations than infested plants treated with buprofezin, cypermethrin or deltamethrin (F_6,170_ = 6.703, *p* < 0.001) (Table S1). Planthopper densities and biomass densities were highest on plants that had received a single, pre-infestation application, and were lowest on plants that received three applications (density: F_2,170_ = 4.494, *p* = 0.012; biomass density: F_2,170_ = 4.253, *p* = 0.016) (Figure 1, Table S1; see also linear contrasts in Table S2). There was a significant variety × application frequency interaction for biomass density because of a higher biomass of BPH on IR64 plants treated with one and two applications, but similar densities on both varieties treated with three applications (F_2,170_ = 4.942, *p* = 0.008; Figure 1). There were also significant variety × treatment interactions because of higher mortality of cypermethrin-treated and deltamethrin-treated IR64 plants compared to similarly treated IR62 plants (F_5,170_ = 3.705, *p* = 0.003: Table S1) and higher densities of BPH on carbofuran-treated IR62 than carbofuran-treated IR64, but higher densities of BPH on cartap hydrochloride-treated and cypermethrin-treated IR64 than on similarly treated IR62 (F_5,170_ = 3.241, *p* = 0.008) (Table S1). All other interactions were non-significant.

**Figure 2:**
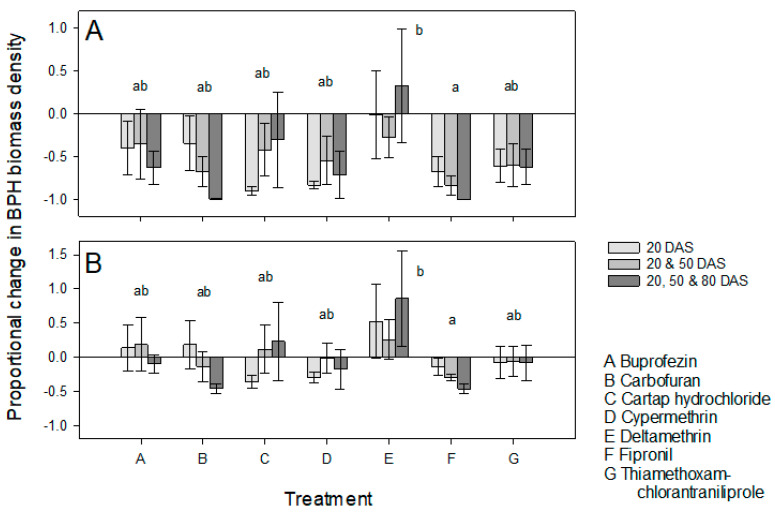
Proportional changes in BPH biomass densities relative to non-treated, BPH-infested IR64 due to (**A**) host plant resistance combined with insecticide applications and (**B**) the estimated insecticide contribution alone. Plants received one (light gray), two (medium gray) or three (dark gray) applications of seven insecticides (x-axis, A–G) and were infested with four and two gravid BPH at 40 and 60 DAS, respectively. Positive numbers indicate proportional increases in BPH biomass density, negative numbers are proportional declines. Standard errors are presented (*n* = 5 blocks). Lowercase letters indicate homogenous insecticide groups based on Tukey pairwise comparisons (*p* ≤ 0.05).

The combined effect of host resistance and insecticide treatments was greatest for IR62 combined with fipronil and was significantly better than IR62 with deltamethrin (F_6,84_ = 2.393, *p* = 0.035) (Figure 2A). Three applications of deltamethrin resulted in higher mean biomass densities of planthoppers on IR62 compared to non-treated IR64 plants. There was no significant effect of application frequency on biomass density (F_2,84_ = 0.021, *p* = 0.979) and no significant interaction (Figure 2A). Similarly, the estimated effect of fipronil alone resulted in the greatest reductions in biomass densities on plants and was significantly greater than the impact of deltamethrin (F_6,84_ = 2.179, *p* = 0.053). There was no significant effect of application frequency (F_2,84_ = 0.019, *p* = 0.981) or the interaction (Figure 2B). In a number of cases, the estimated mean effects of the insecticides, without the resistance of IR62, resulted in higher mean BPH biomass densities compared to infested IR64 plants (i.e., buprofezin, carbofuran, cartap hydrochloride, and deltamethrin in Figure 2B).

### 3.2. Effects of Host Resistance and the Frequency of Insecticide Applications on Plant Growth

Details of the timing of harvest and the anatomy of infested plants (excluding grain) are presented in Table S3. Only plant height significantly improved after treatments with insecticides (Table S3). However, there was also a significant variety × control interaction because of similar heights of treated IR62 and IR64 plants, but shorter non-treated IR64 plants compared to non-treated IR62 plants. There were also several significant interactions between the control factor and variety because of similar values for treated and non-treated IR62, but lower values for non-treated IR64 compared to treated IR64 (i.e., harvest time, root length, number of panicles, above ground biomass and root biomass: Table S3). Plant growth parameters (i.e., treatment effect for height, root length, panicle number and above and below-ground biomass, *p* ≤ 0.05: Table S3) were often lower on buprofezin and deltamethrin-treated plants because of generally high BPH biomass densities on the treated plants compared to plants treated with the remaining insecticides. The effects were often greater for treated IR64 plants (i.e., variety × treatment interaction for root length, panicle number, above and below ground biomass, *p* ≤ 0.05: Table S3).

Details of grain yield are presented in Table 2. There was no significant effect of insecticide treatments compared to non-treated controls on grain yield. However, there was a significant control × variety interaction because of yield improvements on treated IR64, but similar yields on control and insecticide-treated IR62 plants. This also produced a significant variety effect on yield. Across treatments, plants treated with carbofuran produced higher yields than plants treated with buprofezin, cypermethrin or deltamethrin. There was a significant variety × treatment interaction because of similar yields on carbofuran, fipronil and thiamethoxam + chlorantraniliprole-treated IR62 and IR64, but lower yields on IR64 treated with the remaining insecticides (Table 2). Only carbofuran (2 or 3 applications) and fipronil (1, 2, or 3 applications) improved rice yields over controls. In both cases, significant variety × treatment interactions resulted from lower yields of non-treated IR64 compared to non-treated IR62, but similar yields on both varieties when treated with carbofuran or fipronil (Figure S2, Table S2).

Higher yields of treated plants were largely due to higher proportions of filled grain (control factor: F_1,72_ = 5.405, *p* = 0.023), with a greater difference between proportions filled in treated and non-treated IR64, than in corresponding IR62 plants producing a significant control × variety interaction (Table 2). Similarly, the numbers of filled grain were lower in control IR64 plants compared to treated plants (IR62 and IR64) and control IR62 plants, resulting in a significant interaction between the control factor and variety. More grain was produced by IR62 plants and on carbofuran, fipronil and thiamethoxam + chlorantraniliprole-treated plants, with a significant variety × treatment interaction because of similar grain numbers for IR62 and IR64 under these three treatments, but lower grain numbers on IR64 than IR62 for the remaining treatments (Table 2). Grain size was smaller on deltamethrin-treated plants, but there was also a variety × treatment interaction because of similar grain sizes on thiamethoxam + chlorantraniliprole-treated plants, but smaller grain size on IR62 plants compared to IR64 plants under the remaining treatments.

There were no apparent phytotoxic effects on insecticide-treated plants that were not infested with BPH (Table S4). Among the non-infested plants, IR62 plants were harvested earlier, produced more tillers and panicles, and had longer roots than IR64. IR64 plants had greater above-ground biomass. IR62 plants produced more, but smaller grain, resulting in higher yields than in IR64. Although not statistically significant, some of the differences between yields in the two varieties may be due to insecticide effects, particularly the effects of cypermethrin and deltamethrin in reducing yields below the mean yields of the non-treated IR64 controls (Table S4).

### 3.3. Effects of Application Time and Host Resistance on BPH Populations

**Figure 3:**
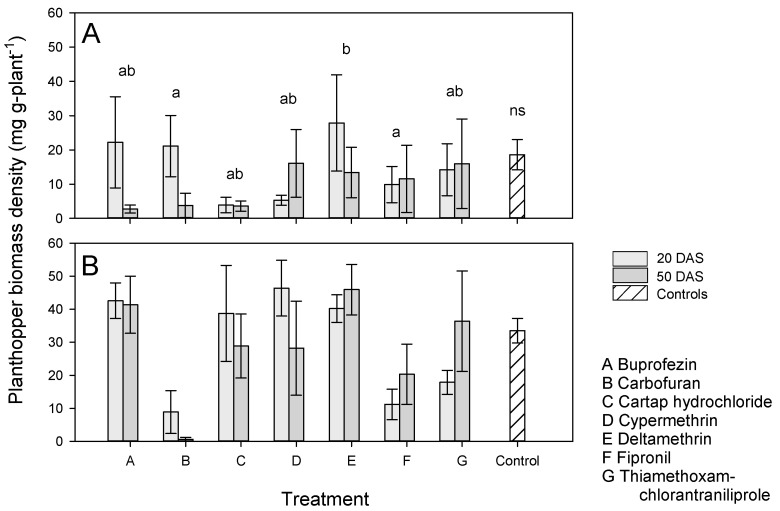
Biomass densities of BPH on (**A**) IR62 (resistant) and (**B**) IR64 (susceptible) treated with one of seven insecticides (x-axis, A–G) applied at 20 DAS (light gray) or 50 DAS (dark gray). Planthopper biomass on non-treated, but infested control plants are indicated by hatched bars. All plants were infested with four gravid BPH at 40 DAS and two further gravid BPH at 60 DAS. Standard errors are indicated (*n* ≤ 5 blocks). Lowercase letters indicate homogenous treatment groups based on Tukey pairwise comparisons (*p* ≤ 0.05); ‘ns’ indicates no significant difference between control and insecticide-treated plants (*p* ≤ 0.05).

Insecticide-treated and control plants had similar BPH densities (F_1,123_ = 1.301, *p* = 0.256) and biomass densities (F_1,123_ = 0.543, *p* = 0.462) (Figure 3, Table S5); however, a higher proportion of control plants died during the experiment (F_1,123_ = 3.999, *p* = 0.048: Table S5). The densities (F_1,123_ = 7.385, *p* = 0.007: Table S5) and biomass densities (F_1,123_ = 19.816, *p* < 0.001) of BPH were higher on IR64 compared to IR62 (Figure 3). Plant mortality was also higher for infested IR64 plants (F_1,123_ = 22.142, *p* < 0.001: Table S5). Plants treated with carbofuran and fipronil had a lower biomass density of BPH than deltamethrin-treated plants (F_6,123_ = 2.674, *p* = 0.017) and more deltamethrin-treated plants died compared to plants treated with other insecticides (F_6,123_ = 2.501, *p* = 0.033: Table S5). There were significant variety × treatment interactions because of similar biomass densities (F_5,123_ = 2.479, *p* = 0.035) on carbofuran and fipronil-treated plants of both varieties, but a higher biomass density of BPH on IR64 plants treated with the other pesticides, and lower BPH biomass densities (F_5,123_ = 3.986, *p* = 0.002) on fipronil-treated IR62 and IR64, but higher densities on IR64 treated with all other insecticides. Only plants treated with carbofuran at 50 DAS had significantly lower biomass densities of BPH than non-treated controls (Figure S3, Table S6). There was a significant variety × application time interaction because of similar BPH densities on plants treated at 20 DAS, but higher densities on IR64 plants compared to IR62 plants treated at 50 DAS (F_1,123_ = 5.311, *p* = 0.023: Table S5), this was largely due to apparent synergies between IR62 resistance and buprofezin and cartap hydrochloride at 50 DAS.

**Figure 4:**
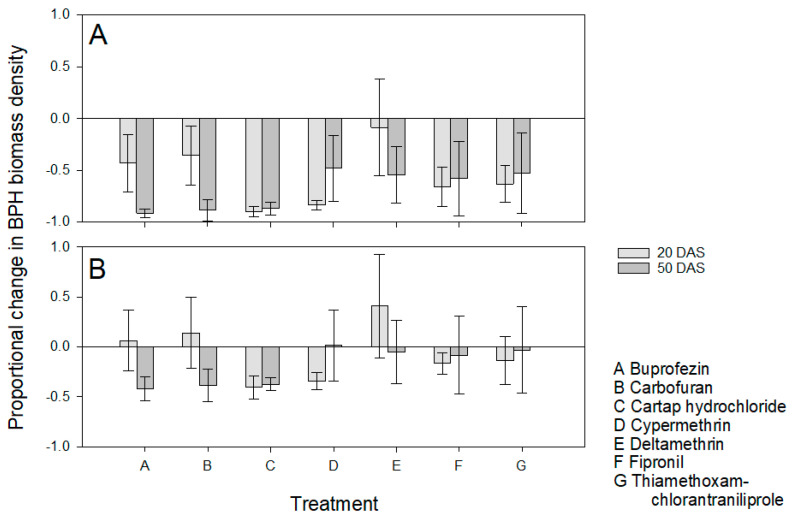
Proportional changes in BPH biomass densities relative to non-treated, BPH-infested IR64 due to (**A**) host plant resistance combined with insecticide applications and (**B**) the estimated insecticide contribution alone. Plants received applications of one of seven insecticides (x-axis, A–G) at 20 DAS (light gray) or 50 DAS (dark gray). All plants were infested with four and two gravid BPH at 40 and 60 DAS, respectively. Positive numbers indicate proportional increases in BPH biomass density, negative numbers are proportional declines. Standard errors are presented (*n* ≤ 5 blocks). Lowercase letters indicate homogenous insecticide groups based on Tukey pairwise comparisons (*p* ≤ 0.05).

There was no significant effect of different insecticide treatments combined with host resistance (F_6,56_ = 0.856, *p* = 0.533: Figure 4A) or without resistance (F_6,56_ = 0.660, *p* = 0.682: Figure 4B) in reducing BPH biomass densities compared to infested control IR64. Although early applications of buprofezin, carbofuran and deltamethrin produced a mean increase in BPH biomass compared to IR64 controls, there was no statistically significant effect of application time on the combined effects of insecticides and resistance (F_1,56_ = 0.896, *p* = 0.348) or the estimated effects of insecticides alone (F_1,56_ = 0.678, *p* = 0.414) and interactions were also non-significant (Figure 4).

### 3.4. Effects of Application Time and Host Resistance on Plant Growth

Details of the growth and development of infested plants are presented in Table S7, with details of yields and grain production presented in Table 3. Because of higher mortality, control plants were harvested earlier than insecticide-treated plants (Table S7). Higher densities of BPH on IR64 compared to IR62 reduced harvest times, tiller and panicle numbers, height, above ground biomass and root biomass (Table S7). Harvest times were longest and the numbers of panicles were highest for carbofuran and fipronil-treated plants, and significantly longer and higher than deltamethrin-treated plants (Table S7). Plants treated at 50 DAS were shorter and had a lower biomass than plants treated at 20 DAS (Table S7). There were a number of significant interactions: IR64 plants treated with buprofezin, cypermethrin or deltamethrin had shorter harvest times and fewer panicles than IR62 plants that received the same treatments; plants under all other treatments were similar. Late applications of fipronil and thiamethoxam + chlorantraniliprole reduced above and below ground biomass, but the same application time effect was not apparent for plants treated with the remaining insecticides (Table S7).

Yields were lower from control plants; however, yields from infested IR62 controls were similar to yields from treated IR64 plants, producing a significant control × variety interaction (Table 3). Yields were higher on IR62, highest on carbofuran-treated plants, and lowest on deltamethrin-treated plants. Similar yields on carbofuran, fipronil or thiamethoxam + chlorantraniliprole-treated IR62 and IR64 plants, but lower yields on IR64 under the remaining treatments, produced a significant variety × treatment interaction. Compared to non-treated controls, yields were higher on plants treated with carbofuran at 50 DAS, and on plants treated with either cartap hydrochloride or fipronil at 20 DAS (Figure S4, Table S6). Higher yields in IR62 were due to more filled grain produced and lower proportions of unfilled grain (Table 3). Despite planthopper damage, IR64 continued to produce larger grain where plants survived. The numbers of filled grain were similar on IR62 and IR64 treated with carbofuran or fipronil but were lower in IR64 plants under all other treatments (variety × treatment interaction). The proportion of grain that was filled was similar for IR62 and IR64 only when treated with fipronil (variety × treatment interaction: Table 3). The time of treatment had no effect on grain production (Table 3).

**Figure 5:**
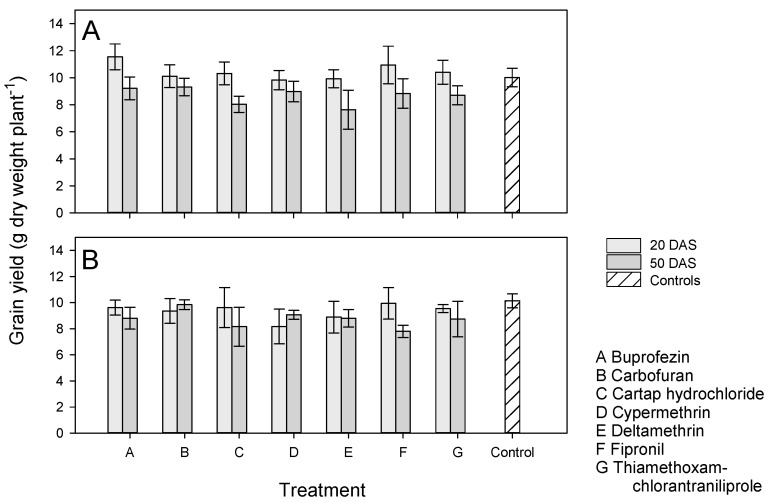
Yields of (**A**) IR62 (resistant) and (**B**) IR64 (susceptible) plants treated with one of seven insecticides (x-axis A–G) at 20 DAS (light gray) or 50 DAS (dark gray). The plants were not infested with planthoppers. Control, non-treated plants are indicated by hatched bars. Standard errors are presented (*n* ≤ 5 blocks). See also Table S8.

Non-infested IR62 plants reached maturity earlier, had higher tillering and had higher root biomass than non-infested IR64 plants; IR64 plants had longer roots and greater above ground biomass (Table S8). Treatment had no significant effect on harvest time, tiller number, plant height, root length, panicle number, above ground weight or root weight of non-infested IR62 and IR64 plants (Table S8). IR62 plants treated with buprofezin had shorter roots than IR64 plants under the same treatment (producing a significant variety × treatment interaction). Plants that received applications at 50 DAS had lower above ground biomass (Table S8). Non-infested IR62 plants produced more, but smaller, grain than non-infested IR64 plants, but grain filling and yields were similar for the two varieties (Table 3). Insecticide treatments had no effect on yields and grain production compared against each other and compared to control, non-treated plants (Figure 5). However, the final yields of treated plants declined where applications were made at 50 DAS (F_1,123_ = 11.459, *p* ≤ 0.001: Figure 5). This was due to lower levels of grain filling in plants treated at 50 DAS compared to plants treated at 20 DAS (Table S8). There was also a significant variety × application time interaction because of a reduction in grain size on IR64 plants treated at 50 DAS (Table S8).

## 4. Discussion

During analysis of our results, we focused on possible outcomes of interactions between insecticides and resistant rice plants as outlined in Table 1. In general, our results indicated IR62 as effective in reducing the buildup of BPH populations and biomass, and, therefore, in reducing consequent damage and maintaining rice yields (Figure 1 and Figure 3, Table 2 and Table 3). We selected seven insecticides without a priori information regarding their effectiveness; of these only carbofuran, fipronil and cypermethrin significantly reduced BPH biomass densities or increased yields in our experiments. Cypermethrin was effective only when applied three times (Figure 1). Thiamethoxam + chlorantraniliprole also tended to improve yields on infested IR64 plants (Figure S2). None of the insecticides caused BPH resurgence in the experiments; however, there was a tendency for deltamethrin to increase biomass densities, particularly after repeated applications (Figure 2 and Figure 4). We now consider the possible effects of combining host resistance with insecticides for the management of BPH.

### 4.1. Potential Synergies

Our main prediction was that insecticides would function synergistically with host resistance to reduce BPH populations below that of either using insecticides alone, or of host plant resistance alone; thereby maintaining or improving the yields of infested rice. Such synergies would arise where resistance weakens the target insects, making them more susceptible to insecticidal toxins [34]. Resistance in IR62 has been associated with smaller individuals and slower growth of BPH, lower fecundity and egg-laying and consequently low population growth rates [37,45]. As planthopper densities increase, the detrimental effects of resistance also increase, (i.e., causing higher mortality of nymphs at higher densities: [47]) producing a stabilizing effect. In our experiments, BPH biomass densities on insecticide-treated IR62 were often lower than on similarly treated IR64 because the insecticides were largely ineffective. However, for those products and application times that functioned relatively well in the experiments (i.e., carbofuran, fipronil and cypermethrin), insecticide-related reductions in BPH biomass density on IR62 were never significantly greater than on treated IR64 (Figure 1 and Figure 3). Nevertheless, there were some tendencies (statistically non-significant) toward synergies as indicated in Table 4. For example, early applications of cartap hydrochloride and cypermethrin consistently reduced BPH biomass densities on IR62, but not on IR64 (Figure 1 and Figure 3), and cartap hydrochloride or buprofezin applied at 50 DAS reduced biomass densities on IR62 compared to IR64 (Figure 3). We are unaware of the mechanisms underlying these possible synergies, but the effects likely relate to interactions between the chemical toxins and the hosts’ antixenotic defenses where the insecticides were applied prior to BPH infestation and to increasing BPH susceptibility to insecticidal toxins when applied after infestation.

Our experiments largely indicated that applying insecticides to IR62 results in a redundancy of management actions. Where insecticides are ineffective against BPH, or where applying insecticide to IR64 resulted in similar biomass densities to non-treated IR62 (i.e., thiamethoxam + chlorantraniliprole), then the applications can be deemed redundant in the context of our experiments. Such redundancies will result in profitability losses for farmers at larger scales [25]. For effective insecticides, such as carbofuran, fipronil and repeated applications of cypermethrin, BPH densities were similar on treated IR62 and IR64 plants, suggesting that, in these cases, host resistance was redundant (Table 4). Redundancies in host resistance should be avoided in farmers’ fields because they signify unnecessary exposure of resistance genes to evolving BPH populations. Redundancies in insecticides may be less problematic, particularly if the insecticides are effective against other rice pests, such as stemborers. However, where the insecticides target BPH, such as in the case of buprofezin, insecticide redundancy results in unnecessary costs and environmental contamination.

In our experiments, the frequency of applications affected the success of insecticides in reducing BPH densities (Figure 1). In general, three applications were better than one or two applications in controlling BPH on IR64; but often not on IR62 (i.e., buprofezin, cartap hydrochloride, cypermethrin, and thiamethoxam + chlorantraniliprole, Figure 1). This implies that multiple applications of insecticide products to control BPH in fields of resistant rice will simply augment profitability losses. Based on the results from our first experiment, prophylactic applications of carbofuran and fipronil, although these products were the most effective in our study, when applied prior to BPH infestations at 40 or 60 DAS did not function to significantly reduce BPH populations on the resistant variety (Figure 1 and Figure 2). In a previous study, carbofuran had no effect on BPH oviposition or nymph survival when applied prior to infesting plants with gravid BPH females or early instar nymphs, respectively [24]. In the present study, carbofuran reduced BPH densities below IR62 controls only when applied three times and after infestations had ceased (i.e., at 80 DAS); but carbofuran effectively reduced BPH biomass on IR64 even when applied on a single occasion at 20 DAS (before infestation). In contrast, fipronil did reduce both oviposition and nymph survival on both varieties when plants were treated 3 [24] or 10 days (this study) prior to infestations. Buprofezin, cypermethrin, cartap hydrochloride and deltamethrin all failed to reduce BPH densities and/or biomass densities on IR64 when applied before planthopper infestations. Furthermore, applying these chemicals prior to BPH infestation often resulted in a higher biomass of BPH on treated IR62 than on control plants. Because BPH and other planthoppers are highly mobile [4], gravid females will disperse between rice plants and fields throughout crop development. Our results suggest that, whereas certain insecticides might be effective against nymphs or are directly toxic to free-living BPH, the same chemicals could also indirectly stimulate oviposition by non-treated females. Evidence suggests that this occurs after early applications of buprofezin [24,48] and possibly cartap hydrochloride [24].

Under controlled conditions and without BPH infestations, IR62 and IR64 both produced about 10 g of filled grain per plant. When infested with BPH, IR62 produced between 4.47 and 5.66 g, and IR64 produced 0.43 g without the application of chemical insecticides (and in the case of our experiments, without natural enemies). Although there were no statistically significant differences between the yields of control and treated plants, the yields of IR62 plants were often higher on carbofuran and fipronil-treated plants than on the infested controls (i.e., yields after carbofuran at two or three applications = > 7 g; yields after fipronil at one, two or three applications = > 7 g: Table 2, see also Figure S2). Some of the higher yields of carbofuran-treated plants may be due to direct chemical effects on the plants; for example, in a screenhouse experiment, Horgan et al. (2021) [24] found that IR62 plants treated with carbofuran had slightly higher yields than non-treated controls, despite similar BPH densities on control and treated plants. A number of studies have indicated that carbofuran stimulates rice growth and yields [49,50,51] and stimulates the availability of nitrogen in the rhizosphere [52]. In our second experiment, all of the insecticide treatments produced higher yields on BPH-infested IR62 plants compared to the control plants (i.e., >4.47 g) (Table 3). Although the yields of treated and non-treated plants were not statistically different, if the results from our pot experiments are scalable to farmers’ fields, then such insecticide-related yield increases could represent an economically significant effect on crop productivity. Furthermore, in many cases the yield advantages apparent on treated plants in our study were independent of any insecticide-induced enhancement of BPH fitness (i.e., cypermethrin and deltamethrin). However, we caution that, while useful to elucidate mechanisms of interaction between plants, BPH and insecticides, pot experiments are subject to high variance and there were several inconsistencies in the results from our two experiments that should be noted. For example, compared to non-treated controls, a single application of carbofuran at 20 DAS in our first experiment resulted in lower yields of IR62; however, in our second experiment, the same treatment resulted in higher yields of IR62. Finally, in a larger, more representative experiment conducted in a screenhouse where plant roots were uninhibited by pot size, applications at 20 and again at 50 DAS resulted in lower yields of IR62 treated with all products except carbofuran, and yield declines due to cypermethrin and fipronil were statistically significant [24].

### 4.2. Potential Antagonisms

We predicted that pyrethroids would stimulate planthopper population growth. We based our prediction on a number of previously published studies that have demonstrated deltamethrin and cypermethrin-induced resurgence of BPH under controlled and field conditions [9,11,18,53,54,55,56,57]. Both deltamethrin and cypermethrin are still commonly used in rice fields despite evidence concerning their association with BPH outbreaks [9,11,56]. More recently, cases of herbivore resurgence in rice have also been associated with some of the other insecticides that we used in our experiments (i.e., buprofezin [16,48] and carbofuran [51,56]).

In our experiments, we did not observe statistically higher populations of BPH on treated plants compared to controls; however, among treated plants, mean BPH densities and biomass densities were often higher on infested IR62 plants that were treated with deltamethrin, with the most obvious effect where deltamethrin had been applied three times (Figure 1A, Table 1). The tendency for three applications of deltamethrin to increase BPH biomass densities on IR62 to a greater extent than on IR64 suggests that the insecticide was antagonistic to the resistance of IR62 (Figure 1, Table 4). Deltamethrin is a recognized resurgence-causing insecticide. The insecticide may have had a higher impact on free-living BPH that were feeding on IR62 compared to IR64—possibly because of changes to insect behaviors on the resistant plants (i.e., lower mobility, reduced feeding, or increases in xylem-feeding [58]). Part of the mechanism underlying deltamethrin-induced resurgence seems to derive from a large decline in antixenotic resistance [11,34,56]. At lower application frequencies, and particularly when deltamethrin was applied only once at 50 DAS, resistance tended to counter related increases in BPH biomass density (Figure 3, Table 4). Therefore, host resistance could stabilize BPH populations by reducing any stimulatory effects of prophylactic, or otherwise untimely insecticide applications, but this effect may be limited to infrequent applications. This stabilizing effect has been referred to as ‘buffering’ in a previous paper. Buffering counters the negative effects of insecticides and is therefore useful to prevent secondary outbreaks of BPH or other targets of resistance [24]. However, buffering also masks the negative effects of insecticides, potentially promoting a higher insecticide use than is required and sustaining the sale and use of resurgence-causing insecticides. For example, Gallagher et al. [59] observed that the adaptation by BPH in Indonesia to rice with the *bph2* gene led to widespread outbreaks of the planthopper, not because of the loss in gene functioning, but because the negative effects of resurgence-causing insecticides were no longer countered.

### 4.3. Phytotoxicity

The application of toxic chemicals to living plants can cause changes to plant growth and development, particularly at seedling stages [60,61,62,63]. Optimally, such changes will be of short duration. However, in some cases, pesticides can result in sustained changes to plants that affect functionality. For example, rice treated with deltamethrin, methyl parathion or quinalphos has higher sugar and protein contents and lower concentrations of defensive phenols [15,22]. These changes have been associated with an increased susceptibility of rice to herbivores [10,15]. Phytotoxicity can also affect plant biomass and yields [24,34,56]. In a previous study with IR62 and IR64 grown in a screenhouse, cypermethrin and fipronil were shown to directly reduce yields [24]. In the present study, we predicted that plants that received insecticide treatments during booting (50 DAS) and grain filling, would exhibit phytotoxic responses, that might include reductions in grain yields.

In our first experiment, we found no evidence of phytotoxicity to rice plants without BPH. Plants treated with one, two or three applications of each insecticide had similar yields to non-treated controls, despite applying the final treatment only 10–15 days before harvest (Table S4). However, despite a lack of statistical significance, plants that had received three applications generally produced less grain than the corresponding non-treated controls. If these effects are scaled to field levels, then such reductions in yields could represent significant economic penalties for farmers who unnecessary apply insecticides at late crop stages (but see caveats of pot experiments indicated above). In our second experiment, the direct negative effects of late insecticide applications (i.e., <45 days before harvest) were more apparent. In the experiment, although there was no difference between treated and control plants, there was a significant effect of application time on above ground biomass (Table S8) and final plant yields (Figure 5). Late applications of buprofezin, cartap hydrochloride or fipronil also consistently reduced yield to below controls in the experiment (*p*-values ranged from 0.067 to 0.080: Table 4). Reductions in yields were due to lower grain production following late applications and a reduction in the proportions of grain that were filled (Table S6). Even when treated with carbofuran, which stimulates plant biomass production, late applications resulted in comparatively slower growth and lower yields on IR62 (Table S8, Figure 5). These results indicate that late-stage applications of insecticides to rice should be avoided. Late applications are sometime made to control grain-feeding insects such as rice bug (*Leptocorisa* spp.) that reduce grain quality but not grain yields [64,65]. Such unnecessary applications to rice at late crop stages might result in lower rice yields and losses to the profitability of rice farms, especially when applied to fields of resistant rice. Further research under more natural conditions is warranted to investigate this phenomenon further.

### 4.4. Implications for BPH Management

Our results indicate that insecticide applications to rice for the control of BPH must be made following monitoring of herbivore risks. At tropical latitudes, BPH and other insects have multiple generations during each crop season [4]. Each generation includes dispersing adults that move between plants and fields. Many of the products that we examined directly reduced BPH densities or biomass densities and maintained rice yields. However, prophylactic applications run the risk of applying insecticides before the initiation of infestations. In many cases, such applications, probably because of phytotoxic effects on the plants, can stimulate population growth in BPH and other rice herbivores [9,10,15,16,17,18,19,22,23,24,34,53,54,56,66,67,68]. Because the insecticides also affect the natural enemies of rice herbivores (i.e., buprofezin [69,70,71,72,73], carbofuran [74,75], cartap hydrochloride [76], cypermethrin [74,77], deltamethrin [71,78], fipronil [72,76,79,80] and thiamethoxam + chlorantraniliprole [70,72,73,81]), and thereby reduce the regulation services of the rice ecosystem, such insecticide-induced BPH populations can continue to grow. This could lead to successive insecticide treatments in response to higher insecticide-induced herbivore densities and further promotes a lock-in to insecticide use.

Host plant resistance buffers against the undesirable effects of insecticides and promotes stability in rice fields; however, we noted variability in the results of our replicated experiments (i.e., applications at 20 DAS in our first and second experiments), and differences between the results of our experiments with potted plants and the results of a previous study with plants in soil-filled concrete bays. Such variability may derive from weather effects on the functioning of resistance or insecticides [82], as well as weather and density effects on planthoppers [47,83]. Ecosystem-based resistance to BPH as provided by natural enemies might be more resilient of changes to climate or other field conditions than either host resistance or insecticides [84]. Only three of the products (carbofuran, fipronil and cypermethrin) that we examined significantly reduced final BPH biomass on IR64 in our experiments, thiamethoxam + chlorantraniliprole also tended to reduce biomass densities and increase IR64 yields (Figure 1B and Figure 3B). Cartap hydrochloride and cypermethrin also tended to reduce BPH densities on IR62 when applied before infestation and buprofezin and cartap hydrochloride reduced biomass densities on IR62 when applied once after infestation (Figure 3). However, all of these chemicals directly reduced rice yields when applied at late growth stages (Figure 5), and in a previous study [24] cypermethrin and fipronil reduced yields below that of non-treated controls. Under field condition, natural enemies prevent BPH and other herbivores from attaining high densities on non-treated rice, such that the densities we report for control plants (IR62 and IR64) are not normally encountered in well managed, biodiverse rice fields [67,84,85]. Based on the potential negative effects of unnecessary insecticide applications, actions should be taken to counter insecticide marketing that promotes prophylactic applications without any compensation to farmers for consequent outbreaks or yield losses.

## 5. Conclusions

We found evidence that resistance with single applications of some insecticides gives better control of BPH than either method alone. Resistance also buffered against resurgence by deltamethrin, but the same insecticide was antagonistic to the host’s resistance after repeated applications. Each of these effects was weak in our experiments. Many of the insecticides were ineffective in our experiments and their use for BPH control on resistant rice was therefore redundant. Meanwhile, the combination of effective insecticides (carbofuran, fipronil) with resistance, or multiple applications of less effective insecticides (cartap hydrochloride, cypermethrin) resulted in a redundancy of resistance and a loss of synergies. Among the products we examined, three products (carbofuran, fipronil and cypermethrin) reduced BPH densities and biomass densities when applied after planthoppers had colonized the rice plants. However, at least two of these products, carbofuran and fipronil, have severe impacts on farmers and pesticide applicators or on wildlife, and cypermethrin and fipronil can also directly reduce rice yields [24,80,86,87,88,89]. Because of the vagaries of insecticides, that depend on the frequency and timing of applications as well as interactions with the host variety and environment, farmers should ensure that natural enemies are conserved in their rice fields and that ecosystem resilience is maintained by planting resistant rice, but avoiding insecticide use.

## Figures and Tables

**Table 1:** Possible outcomes from applying host plant resistance and insecticide applications, either alone or in combination, on brown planthopper (BPH) densities and rice yields. Tests and test consequences are indicated based on experiments conducted in the present study with IR62 and IR64 as resistant and susceptible rice varieties, respectively.

Categories of Outcome ^1^	Outcome Criteria	Tests	Test Consequences ^3^
Resistance effective	BPH densities on resistant rice are lower than densities on susceptible rice; yields are higher on resistant rice	Comparison of final planthopper densities and grain yields on BPH-infested IR62 and IR64	Significantly lower BPH densities and/or higher yields on IR62
Insecticide effective ^1^	BPH densities lower on insecticide-treated rice than on non-treated rice; yields are higher on treated rice	Comparison of final planthopper densities on insecticide-treated and non-treated IR62 and/or IR64 infested with BPH	Significantly lower BPH densities on treated plants
Insecticide resurgence-causing ^1^	BPH densities higher on insecticide-treated rice than on non-treated rice	Comparison of final planthopper densities on insecticide-treated and non-treated IR62 and/or IR64 infested with BPH	Significantly higher BPH densities on treated plants
No benefit (redundant use of insecticide)	Applying insecticide to resistant rice produces no reduction in BPH densities or no increase in rice yields	Comparison of final planthopper densities and grain yields on insecticide-treated and non-treated IR62 infested with BPH	No significant difference between BPH densities, or yields on insecticide-treated and non-treated IR62
No benefit (redundant exposure of variety)	Applying an effective insecticide to resistant and susceptible rice results in similar BPH densities or similar yield increases irrespective of host resistance	Comparison of final planthopper densities and grain yields on insecticide-treated and non-treated IR62 and IR64 infested with BPH	Significant variety × insecticide effect due to lower BPH densities/higher yields on untreated IR62 compared to untreated IR64, but similar densities and yields on insecticide-treated plants irrespective of variety
Insecticides and resistance synergistic ^2^	Applying insecticide to resistant rice reduces BPH densities or increases rice yields more than on non-treated resistant rice and treated susceptible rice	Comparison of final planthopper densities and grain yields on insecticide-treated and non-treated IR62 and IR64 infested with BPH	Significant insecticide and/or variety effects with or without significant interactions depicting lower densities and higher yields on treated IR62 compared to non-treated IR62
Insecticides antagonistic to resistance	Applying insecticide to resistant rice results in higher BPH densities and/or lower yields than on non-treated resistant rice thereby producing similar BPH densities and/or yields on resistant and susceptible rice	Comparison of final planthopper densities and grain yields on insecticide-treated and non-treated IR62 and IR64 infested with BPH	Significant variety × insecticide interaction due to similar densities of BPH and/or yields on treated IR62 and treated IR64, but lower densities and/or higher yields on nontreated IR62 compared to non-treated IR64
Resistance buffers against antagonistic effects	Applying a resurgence-causing insecticide to resistant rice has no effect on BPH densities or rice yields, but the same insecticide on susceptible rice increases BPH densities and/or decreases yields	Comparison of final planthopper densities and grain yields on IR62 and IR64 treated with resurgence-causing insecticide and infested with BPH	Significant variety effect maintained after BPH densities significantly increase on IR64, but not on IR62
Insecticide phytotoxic	Applying insecticide to non-BPH infested resistant or susceptible rice reduces plant vigor, possibly reducing yields	Comparisons of insecticide-treated and non-treated IR62 and/or IR64 plants without BPH infestation	Significant reduction in plant biomass and/or yields
Insecticide stimulates plant development	Applying insecticide to non-BPH infested resistant or susceptible rice increases plant vigor, possibly increasing yields	Comparisons of insecticide-treated and non-treated IR62 and/or IR64 plants without BPH infestation	Significant increase in plant biomass and/or yields

^1^ Note that an effective insecticide reduces BPH densities to maintain yields. Because insecticides can directly stimulate or reduce rice yields, final yields are not considered as a targeted effect of insecticides in this study. ^2^ ‘Synergies’ are equivalent to ‘additive effects’. ^3^ ‘Significant’ implies a statistically significant response at α ≤ 0.05 in the present study.

**Table 2:** Grain production on IR62 (resistant) and IR64 (susceptible) rice varieties infested with brown planthopper and treated with one of seven insecticides one, two or three times in a pot experiment. Numbers are means ± standard errors. For further details concerning infested and non-infested plants see Table S3 and Table S4, respectively.

Variety and Insecticide	Number of Applications	Weight of Filled Grains (g Dry Weight) ^1,2^	Number of Filled Grain ^1^	Proportion of Grain Unfilled ^1^	1000 Grain Weight ^1^
IR62					
Buprofezin	1	6.15 ± 1.98 ^ab^	314.00 ± 96.98 ^ab^	0.25 ± 0.04	19.44 ± 0.67 ^ab^
	2	5.88 ± 2.14	298.40 ± 105.67	0.28 ± 0.10	19.83 ± 1.26
	3	5.93 ± 2.09	342.00 ± 118.25	0.31 ± 0.17	15.73 ± 1.81
Carbofuran	1	4.90 ± 1.84 ^d^	262.60 ± 86.08 ^d^	0.46 ± 0.09	17.76 ± 1.37 ^b^
	2	7.34 ± 1.51	391.60 ± 81.14	0.40 ± 0.12	18.82 ± 0.27
	3	10.32 ± 1.09	557.60 ± 44.02	0.10 ± 0.02	18.34 ± 0.58
Cartap hydrochloride	1	8.62 ± 1.80 ^abcd^	425.20 ± 88.25 ^abcd^	0.24 ± 0.09	20.17 ± 0.53 ^b^
	2	6.14 ± 2.54	317.20 ± 132.04	0.20 ± 0.05	19.39 ± 0.34
	3	5.93 ± 1.99	319.40 ± 102.64	0.32 ± 0.13	18.11 ± 0.93
Cypermethrin	1	7.95 ± 0.83 ^abc^	428.00 ± 34.52 ^abc^	0.38 ± 0.04	18.44 ± 0.56 ^ab^
	2	6.23 ± 2.22	317.20 ± 113.30	0.19 ± 0.05	19.63 ± 0.31
	3	7.51 ± 1.95	411.20 ± 110.99	0.13 ± 0.03	18.44 ± 0.60
Deltamethrin	1	5.22 ± 2.21 ^a^	273.40 ± 114.52 ^a^	0.34 ± 0.02	19.04 ± 0.57 ^a^
	2	3.35 ± 1.96	204.80 ± 108.93	0.34 ± 0.14	13.85 ± 3.34
	3	5.04 ± 2.46	267.40 ± 123.69	0.27 ± 0.05	18.39 ± 0.94
Fipronil	1	6.93 ± 2.09 ^cd^	360.20 ± 98.25 ^cd^	0.31 ± 0.06	18.81 ± 1.56 ^b^
	2	8.03 ± 2.15	427.20 ± 110.36	0.13 ± 0.02	18.69 ± 0.92
	3	6.46 ± 0.82	361.80 ± 41.57	0.28 ± 0.08	17.85 ± 0.71
Thiamethoxam + chlorantraniliprole	1	7.07 ± 3.03 ^bcd^	321.60 ± 133.66 ^bcd^	0.24 ± 0.01	21.81 ± 1.70 ^b^
	2	5.48 ± 2.14	283.40 ± 112.96	0.28 ± 0.15	19.62 ± 0.53
	3	4.49 ± 1.99	245.60 ± 106.75	0.29 ± 0.06	18.10 ± 0.60
Control		5.66 ± 0.61	288.60 ± 22.19	0.44 ± 0.05	17.59 ± 0.66
IR64					
Buprofezin	1	0.61 ± 0.61	42.00 ± 42.00	0.54	14.48
	2	0.71 ± 0.66	3.20 ± 1.96	0.98 ± 0.00	12.50 ± 2.50
	3	2.69 ± 1.50	140.20 ± 72.80	0.38 ± 0.18	18.48 ± 1.70
Carbofuran	1	6.36 ± 2.57	292.40 ± 115.64	0.49 ± 0.18	20.51 ± 1.45
	2	9.46 ± 1.40	447.20 ± 61.31	0.20 ± 0.05	21.02 ± 0.50
	3	8.96 ± 0.48	414.80 ± 20.63	0.21 ± 0.03	21.60 ± 0.46
Cartap hydrochloride	1	2.18 ± 1.35	113.20 ± 66.93	0.61 ± 0.13	18.41 ± 1.05
	2	3.37 ± 1.25	166.80 ± 58.64	0.46 ± 0.11	19.87 ± 0.81
	3	4.18 ± 1.58	217.40 ± 76.18	0.32 ± 0.10	18.75 ± 0.99
Cypermethrin	1	0.00 ± 0.00	0.00 ± 0.00	NG	NG
	2	1.57 ± 0.61	86.00 ± 27.56	0.73 ± 0.04	17.97 ± 4.80
	3	3.28 ± 1.81	178.00 ± 87.05	0.59 ± 0.16	16.02 ± 1.97
Deltamethrin	1	0.06 ± 0.06	0.30 ± 0.30	NG	NG
	2	0.92 ± 0.92	52.40 ± 52.40	0.31	17.63
	3	0.46 ± 0.28	44.00 ± 27.13	0.58 ± 0.18	10.59 ± 0.51
Fipronil	1	6.70 ± 1.56	315.60 ± 72.69	0.36 ± 0.15	20.53 ± 0.85
	2	7.06 ± 1.67	327.20 ± 68.98	0.31 ± 0.12	20.83 ± 0.93
	3	8.24 ± 1.30	389.80 ± 70.96	0.24 ± 0.07	21.44 ± 0.63
Thiamethoxam + chlorantraniliprole	1	5.52 ± 1.68	285.40 ± 89.64	0.40 ± 0.08	19.39 ± 1.06
	2	5.79 ± 1.52	283.20 ± 62.86	0.34 ± 0.10	19.65 ± 0.93
	3	6.90 ± 1.75	353.80 ± 89.53	0.27 ± 0.12	18.69 ± 1.58
Control		0.43 ± 0.19	14.90 ± 9.53	0.77 ± 0.09	15.91 ± 0.93
F-variety (V)		15.938 ***	22.824 ***	12.982 ***	0.555 ns
F-treatment (T)		6.052 ***	5.442 ***	1.554 ns	3.066 **
					
F-applications		0.876 ns	1.537 ns	1.976 ns	0.506 ns
F-V × T		3.669 ***	3.305 **	2.175 ns	2.960 *
F-control (C)		2.508 ns	2.868 ns	5.405 *	2.256 ns
F-C × V		19.985 ***	27.325 ***	11.886 ***	1.579 ns

^1^: ns = *p* > 0.05, * = *p* ≤ 0.05, ** = *p* ≤ 0.01, *** = *p* ≤ 0.005; lowercase letters indicate homogenous treatment (insecticide) groups for IR62 and IR64 based on Tukey pairwise comparisons (*p* ≤ 0.05); see main text for explanations of insecticide × variety effects. NG = no grain produced; means without standard errors are from a single surviving plant. Numerator degrees of freedom for general linear models using the Addelman (1974) [46] method are as follows: variety, 1; treatment, 6; applications, 2; V × T, 5; control, 1; C × V, 1; denominator degrees of freedom are 164 for weight and number of grains, 74 for proportion of grain filled and 75 for 1000 grain weight. Non-significant interactions are not presented. ^2^: Rice yields are also presented in Figure S2 with separate analyses for each insecticide presented in Table S2.

**Table 3:** Grain production on IR62 (resistant) and IR64 (susceptible) rice varieties infested with brown planthopper and treated with one of seven insecticides at 20 or 50 days after sowing in a pot experiment. Numbers are means ± standard errors. For further details concerning infested and non-infested plants, see Table S7 and Table S8, respectively.

Variety and Insecticide	Application Time (Days)	Weight of Filled Grains (g Dry Weight) ^1^	Number of Filled Grain ^1^	Proportion of Grain Unfilled ^1^	1000 Grain Weight ^1^
IR62					
Buprofezin	20	6.15 ± 1.98 ^ab^	260.00 ± 116.23 ^ab^	0.54 ± 0.19 ^abc^	19.45 ± 0.54 ^ab^
	50	8.62 ± 0.80	462.40 ± 29.44	0.17 ± 0.03	18.54 ± 0.82
Carbofuran	20	4.90 ± 1.84 ^b^	220.60 ± 101.38 ^b^	0.65 ± 0.15 ^a^	18.62 ± 0.80 ^b^
	50	8.10 ± 2.05	407.80 ± 103.23	0.33 ± 0.17	19.85 ± 0.22
Cartap hydrochloride	20	8.62 ± 1.80 ^ab^	425.20 ± 88.25 ^ab^	0.24 ± 0.09 ^abc^	20.17 ± 0.53 ^a^
	50	7.04 ± 0.84	358.40 ± 45.42	0.30 ± 0.08	19.75 ± 0.49
Cypermethrin	20	7.95 ± 0.83 ^ab^	428.00 ± 34.52 ^ab^	0.38 ± 0.04 ^abc^	18.44 ± 0.56 ^ab^
	50	4.95 ± 2.00	255.00 ± 104.26	0.56 ± 0.18	19.42 ± 0.37
Deltamethrin	20	5.22 ± 2.21 ^a^	273.40 ± 114.52 ^a^	0.60 ± 0.16 ^c^	19.13 ± 0.32 ^ab^
	50	5.97 ± 1.60	248.20 ± 101.93	0.53 ± 0.19	19.65 ± 0.25
Fipronil	20	6.93 ± 2.09 ^ab^	360.20 ± 98.25 ^ab^	0.45 ± 0.15 ^ab^	18.65 ± 1.22 ^ab^
	50	5.89 ± 1.54	240.80 ± 98.89	0.52 ± 0.20	19.56 ± 0.26
Thiamethoxam + chlorantraniliprole	20	7.07 ± 3.03 ^ab^	321.60 ± 133.66 ^ab^	0.55 ± 0.19 ^abc^	21.69 ± 0.94 ^b^
	50	5.14 ± 2.45	266.20 ± 119.80	0.54 ± 0.19	18.59 ± 0.83
Control		4.47 ± 1.27	220.60 ± 57.87	0.55 ± 0.12	20.76 ± 2.89
IR64					
Buprofezin	20	0.61 ± 0.61	0.90 ± 0.90	1.00 ± 0.00	NG
	50	1.17 ± 0.44	61.00 ± 25.21	0.81 ± 0.08	18.03 ± 0.74
Carbofuran	20	6.36 ± 2.57	292.40 ± 115.64	0.49 ± 0.18	20.50 ± 1.12
	50	6.82 ± 0.72	301.40 ± 31.18	0.16 ± 0.03	22.58 ± 0.25
Cartap hydrochloride	20	2.18 ± 1.35	113.20 ± 66.93	0.76 ± 0.12	18.44 ± 0.60
	50	1.37 ± 0.56	2.40 ± 2.40	0.99 ± 0.01	12.50 ± 0.22
Cypermethrin	20	0.00 ± 0.00	0.00 ± 0.00	1.00 ± 0.00	NG
	50	3.76 ± 1.99	175.80 ± 91.21	0.53 ± 0.20	18.11 ± 2.14
Deltamethrin	20	0.06 ± 0.06	0.20 ± 0.20	1.00 ± 0.00	NG
	50	0.05 ± 0.05	0.20 ± 0.20	1.00 ± 0.00	NG
Fipronil	20	6.70 ± 1.56	315.60 ± 72.69	0.36 ± 0.15	20.53 ± 0.85
	50	3.74 ± 1.45	196.40 ± 80.69	0.41 ± 0.16	19.82 ± 0.85
Thiamethoxam + chlorantraniliprole	20	5.52 ± 1.68	285.40 ± 89.64	0.52 ± 0.14	19.11 ± 0.87
	50	1.97 ± 1.97	88.20 ± 88.20	0.81 ± 0.19	22.46 ± 0.23
Control		0.43 ± 0.19	5.70 ± 5.70	0.97 ± 0.03	16.57 ± 0.23
F-variety (V)		34.375 ***	37.296 ***	20.712 ***	4.689 *
F-treatment (T)		2.337 **	2.084 ns	2.865 **	3.126 **
F-day		0.192 ns	0.308 ns	0.586 ns	0.098 ns
F-V × T		3.189 **	3.455 **	4.588 ***	4.508 ***
F-control (C)		11.218 ***	12.464 ***	1.877 ns	0.592 ns
F-C × V		3.926 *	0.037ns	0.891 ns	6.087 **

^1^: ns = *p* > 0.05, * = *p* ≤ 0.05, ** = *p* ≤ 0.01, *** = *p* ≤ 0.005; lowercase letters indicate homogenous treatment (insecticide) groups for IR62 and IR64 based on Tukey pairwise comparisons (*p* ≤ 0.05); see main text for explanations of insecticide × variety effects. NG = no grain produced. Numerator degrees of freedom for general linear models using the Addelman (1974) [46] method are as follows: variety, 1; treatment, 6; applications, 1; V × T, 5; control, 1; C × V, 1; denominator degrees of freedom are 123 for weight, number of grains and proportion of grain filled, and 77 for 1000 grain weight. Non-significant interactions are not presented.

**Table 4:** Summary of results from combining insecticides with host plant resistance (based on IR62) for the management of the brown planthopper (BPH). Outcomes indicated in bold are explained in Table 1.

Insecticides	Experiment 1 (Application Frequency)			Experiment 2 (Application Timing)		
	BPH Biomass Density	Rice Yield	Phytotoxic Effects	BPH Biomass Density	Rice Yield	Phytotoxic Effects
Buprofezin	Ineffective; insecticide redundant	Ineffective; insecticide redundant	No	Ineffective; insecticide redundant at 20 DAS; synergy—tendency to reduce biomass density at 50 DAS	Ineffective; insecticide redundant	Tendency to reduce yield at 50 DAS
Cabofuran	Effective (2, 3 applications); resistance redundant	Effective (2, 3 applications); resistance redundant	No	Effective (50 DAS)	Tendency toward effective (50 DAS)	No
Cartap hydrochloride	Ineffective; insecticide redundant; synergy—tendency to reduce biomass density on IR62 after 1 application	Ineffective; insecticide redundant	No	Effective; synergy—tendency to reduce biomass density at 20 and 50 DAS	Effective (20 DAS); synergy—tendency to increase IR62 yields at 20 DAS	Tendency to reduce yield at 50 DAS
Cypermethrin	Ineffective (1 or 2 applications); Effective (3 applications); synergy—tendency to reduce biomass density on IR62 after 1 application	Ineffective	No	Ineffective; synergy—tendency to reduce biomass density on IR62 at 20 DAS	Ineffective; synergy—tendency to increase yields on IR62 at 20 DAS	No
Deltamethrin	Ineffective; antagonistic—tendency to increase biomass density on IR62, but not on IR64, after 3 applications	Ineffective	No	Ineffective; buffer—tendency for biomass density to increase on IR64 at 50 DAS, but not on IR62	Ineffective	No
Fipronil	Effective (1, 2, and 3 applications); resistance redundant	Effective (1, 2, and 3 applications); resistance redundant	No	Ineffective; tendency to reduce biomass density at 20 and 50 DAS	Effective (20 DAS); tendency to increase yields at 50 DAS; resistance redundant	Tendency to reduce yields at 50 DAS
Thiamethoxam + chlorantraniliprole	Ineffective; insecticide redundant	Ineffective; tendency to increase yields of IR64; insecticide redundant	No	Ineffective; insecticide redundant	Ineffective; insecticide redundant	No

## Data Availability

The data presented in this study are available on reasonable request from the corresponding author.

## Supplementary Materials

The following supporting information can be downloaded at: https://www.mdpi.com/article/10.3390/insects13020106/s1, Table S1: Planthopper biomass (number per plant) and surviving IR62 and IR64 rice plants treated with one, two or three applications of each of seven insecticides; Figure S1: Biomass density of brown planthopper on IR62 and IR64 rice plants with 0 (control), 1, 2 or 3 applications of each of seven insecticides; Table S2: Results (F-values) from univariate general linear models for planthopper density, biomass density, plant survival and plant yields after 0 (control), 1, 2 or 3 applications of each of seven insecticides; Figure S2: Yields of BPH infested IR62 and IR64 rice plants with 0 (control), 1, 2 or 3 applications of each of seven insecticides; Table S3: Growth parameters for IR62 and IR64 rice varieties infested with brown planthopper and treated with one of seven insecticides in a pot experiment. Plants were treated with one, two or three applications of each insecticide; Table S4: Growth parameters for IR62 and IR64 rice varieties treated with one of seven insecticides. Plants were treated with one, two or three applications of each insecticide; Table S5: Planthopper biomass (number per plant) and surviving IR62 and IR64 rice plants treated at 20 or 50 days after sowing with one of seven insecticides; Figure S3: Biomass density of brown planthopper on IR62 and IR64 rice plants after treatment with each of seven insecticides at 20 or 50 days after sowing and on non-treated controls; Table S6: Results (F-values) from univariate general linear models for planthopper density, biomass density, plant survival and plant yields after treatment with each of seven insecticides at 20 or 50 days after sowing and on non-treated controls; Figure S4: Yields of BPH infested IR62 and IR64 rice plants after treatment with each of seven insecticides at 20 or 50 days after sowing and on non-treated controls; Table S7: Growth parameters for IR62 and IR64 rice varieties infested with brown planthopper and treated with one of seven insecticides at 20 or 50 days after sowing; Table S8: Growth parameters for IR62 and IR64 rice varieties treated with one of seven insecticides at 20 or 50 days after sowing.
