# Supplementary material for: Compatibility of Insecticides with Rice Resistance to Planthoppers as Influenced by the Timing and Frequency of Applications
Source: Insects. 2022 Jan 18;13(2):106. doi: 10.3390/insects13020106 (PMC8880585; doi:10.3390/insects13020106)
Supplement: Supplementary file 1 [file insects-13-00106-s001.zip › insects-1531648-supplementary.pdf]

Insects 2022

Compatibility of insecticides with rice resistance to planthoppers as influenced by the timing and frequency of applications

Finbarr G. Horgan [1,2,3]* and Ainara Peñalver-Cruz [4,5]

1 EcoLaVerna Integral Restoration Ecology, Bridestown, Kildinan, County Cork, T56 P499, Ireland
2 Centre for Pesticide Suicide Prevention, University/BHF Centre for Cardiovascular Science, University of Edinburgh, Edinburgh EH16 4TJ, UK
3 Escuela de Agronomía, Facultad de Ciencias Agrarias y Forestales, Universidad Católica del Maule, Casilla 7-D, 3349001-Curicó, Chile
4 Institut de Génétique, Environnement et Protection des Plantes (IGEPP), Institut National de Recherche pour l'Agriculture, l'Alimentation et l'Environnement (INRAE), Institut Agro, Université de Rennes, CEDEX 49045, Angers, France; ainara.penalver@agrocampus-ouest.fr
5 International Rice Research Institute, Makati 1226, Manila, Philippines
* Correspondence: f.horgan@ecolaverna.org

Supplementary Information

Table S1: Planthopper density (number per g plant) on IR62 and IR64 rice plants treated with one, two or three applications of each of seven insecticides. Results for non-treated controls are also presented. The proportions of plants surviving are also indicated. All plants were infested with four gravid female BPH at 40 DAS and again with two gravid females at 60 DAS. Numbers are means ± SEM

Variety and Insecticide	Number of Applications	Proportion of Plants Surviving[1]	Number of BPH per g of Plant[1]
IR62			
Buprofezin	1	0.60±0.24 [abc]	127.65+38.04
	2	0.60±0.24	164.00+61.64
	3	0.80±0.20	102.19+33.47
Carbofuran	1	0.80±0.20 [c]	320.59±211.83
	2	1.00±0.00	372.38±8.28
	3	1.00±0.00	0.45±0.39
Cartap hydrochloride	1	1.00±0.00 [abc]	43.13±37.06
	2	0.60±0.24	31.58±17.67
	3	0.60±0.24	57.48±42.85
Cypermethrin	1	1.00±0.00 [ab]	72.51±23.82
	2	0.60±0.24	202.24±172.39

	3	0.80±0.20	18.48±16.74
Deltamethrin	1	0.60±0.24 [a]	133.16±21.99
	2	0.40±0.24	94.03±46.42
	3	0.60±0.24	201.89±51.17
Fipronil	1	0.80±0.20 [c]	159.57±146.67
	2	0.80±0.20	9.32±7.07
	3	1.00±0.00	0.00±0.00
Thiamethoxam + chlorantraniliprole	1	0.60±0.24 [bc]	73.36±48.57
	2	0.80±0.20	253.26±172.79
	3	0.60±0.24	236.65±148.09
Control		1.00±0.00	194.58±59.72
IR64			
Buprofezin	1	0.20±0.20	155.19+20.47
	2	0.40±0.24	242.68+49.93
	3	0.40±0.24	70.65+17.83
Carbofuran	1	0.80±0.20	45.75±42.96
	2	0.80±0.20	26.09±10.35
	3	1.00±0.00	0.69±0.31
Cartap hydrochloride	1	0.60±0.24	252.70±41.08
	2	0.40±0.24	518.43±269.90
	3	0.40±0.24	38.82±22.60
Cypermethrin	1	0.00±0.00	163.66±64.40
	2	0.20±0.20	400.66±55.31
	3	0.40±0.24	28.83±19.69
Deltamethrin	1	0.00±0.00	174.06±37.19
	2	0.20±0.20	217.49±44.15
	3	0.40±0.24	205.14±57.14
Fipronil	1	1.00±0.00	75.25±59.37
	2	0.40±0.24	147.27±116.46
	3	1.00±0.00	0.00±0.00
Thiamethoxam + chlorantraniliprole	1	0.80±0.20	142.24±19.83
	2	1.00±0.00	108.67±56.65
	3	1.00±0.00	52.17±31.10
Control		0.20±0.20	251.56±57.39
F-variety (V)		12.049***	0.611ns
F-treatment (T)		7.058***	0.918ns
F-applications		1.721ns	4.494**
F-V×T		3.901***	3.241**
F-control		0.050ns	1.128ns

[1]: ns = P > 0.05, ** = P ≤ 0.01, *** = P ≤ 0.005; lowercase letters indicate homogenous treatment (insecticide) groups for IR62 and IR64 based on Tukey pairwise comparisons (P ≤ 0.05); Numerator degrees of freedom for general

linear models using the Addelman (1974) method are as follows: variety, 1; treatment, 6; applications, 2; V×T, 5; control, 1; denominator degrees of freedom are 170. Non-significant interactions are not presented.

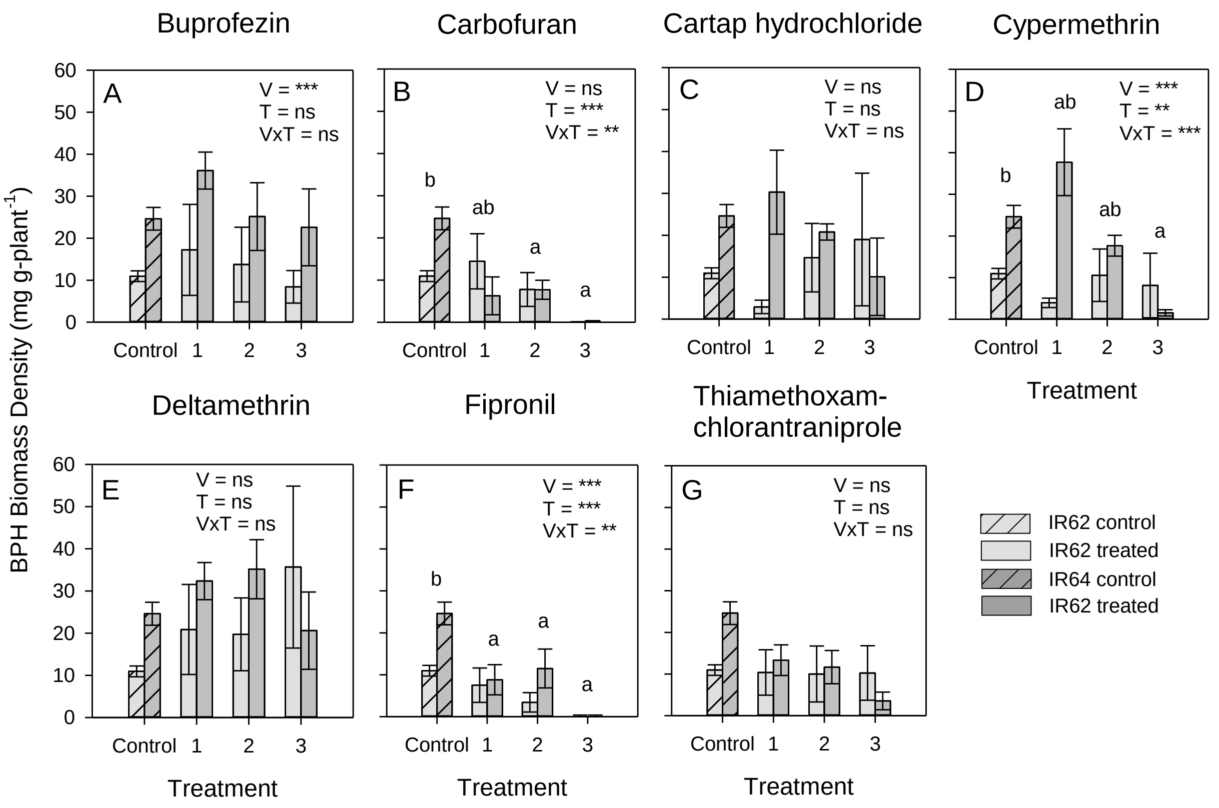

Figure S1: Biomass density of brown planthopper (BPH) on IR62 (resistant) and IR64 (susceptible) rice plants with 0 (control), 1, 2 or 3 applications of A) buprofezin, B) carbofuran, C) cartap hydrochloride, D) cypermethrin, E) deltamethrin, F) fipronil, or G) thiamethoxam-chlorantraniprole. Graphs are redrawn based on data presented in Figure 1 of the main text to highlight control plants versus plants treated with each insecticide. Results of univariate GLMs for each insecticide are indicated as V (variety effect), T (treatment effect, including controls), and VxT (variety x treatment interaction). Ns = not significant, ** = P ≤ 0.01, *** = P ≤ 0.005. Lowercase letters indicate homogenous treatment groups. Standard errors are indicated. For further details of analyses, see Table S2.

Table S2: Results from univariate GLMs for planthopper biomass density (biomass per g plant), and density (number per g plant). Results from analyses of plant survival and plant yields (weight of grain) are also presented. GLMs were conducted separately for each insecticide product. Results from comparative analyses of all seven insecticides are presented in the main text (Figure 1, Table 2)

**Parameter**[1]	**Sources of Variation**[2]	**F-values**[3]						
		**Buprofezin**	**Carbofuran**	**Cartap Hydrochloride**	**Cypermethrin**	**Deltamethrin**	**Fipronil**	**Thiamethoxam + Chlorantraniprole**
Biomass density (mg per g plant)	Variety (V)	10.020***	0.673ns	3.079ns	14.272***	1.120ns	11.474***	1.230ns
	Treatment (T)	0.953ns	9.848***(LC***)	0.108ns	4.607**(LC***)	0.402ns	17.288***(LC***)	2.529ns
	V×T	0.109ns	4.243**	1.751ns	6.399***	1.255ns	3.897**	2.213ns
Density (number per g plant)	Variety (V)	1.407ns	1.747ns	6.258**	2.752ns	3.049ns	0.317ns	0.630ns
	Treatment (T)	0.359ns	0.914ns	1.801ns	5.088**	1.169ns	3.569*(LC***)	0.596ns
	V×T	0.440ns	0.873ns	2.311ns	0.553ns	0.609ns	0.909ns	1.071ns
Plant survival (proportion)	Variety (V)	10.125***	5.738*	6.946**	34.290***	11.340***	5.833*	0.001ns
	Treatment (T)	0.458ns	2.678ns	0.868ns	0.744ns	1.260ns	3.967*	1.152ns
	V×T	0.792ns	3.290*	0.868ns	1.826ns	1.260ns	4.589**	5.070**
Yield (g per plant) - infested	Variety (V)	24.825***	0.559ns	14.587***	135.476***	19.177***	1.738ns	0.469ns
	Treatment (T)	0.330ns	8.668***(LC***)	0.976ns	1.106ns	0.146ns	5.769***(LC***)	1.251ns
	V×T	0.295ns	2.795*	1.044ns	0.816ns	0.439ns	2.790*	1.628ns

1: Means for density and plant survival are presented in Table S1; means for biomass density are presented in Figures 1 and Figure S1; means for yield are presented in Table 2 and Figure S2
2: Degrees of freedom for variety = 1,28; treatment = 3,28; and V×T interaction = 3,28. Block effects are not presented
3: ns = P > 0.05; * = P ≤ 0.05; ** = P ≤ 0.01; *** = P ≤ 0.05; LC = significant linear contrast

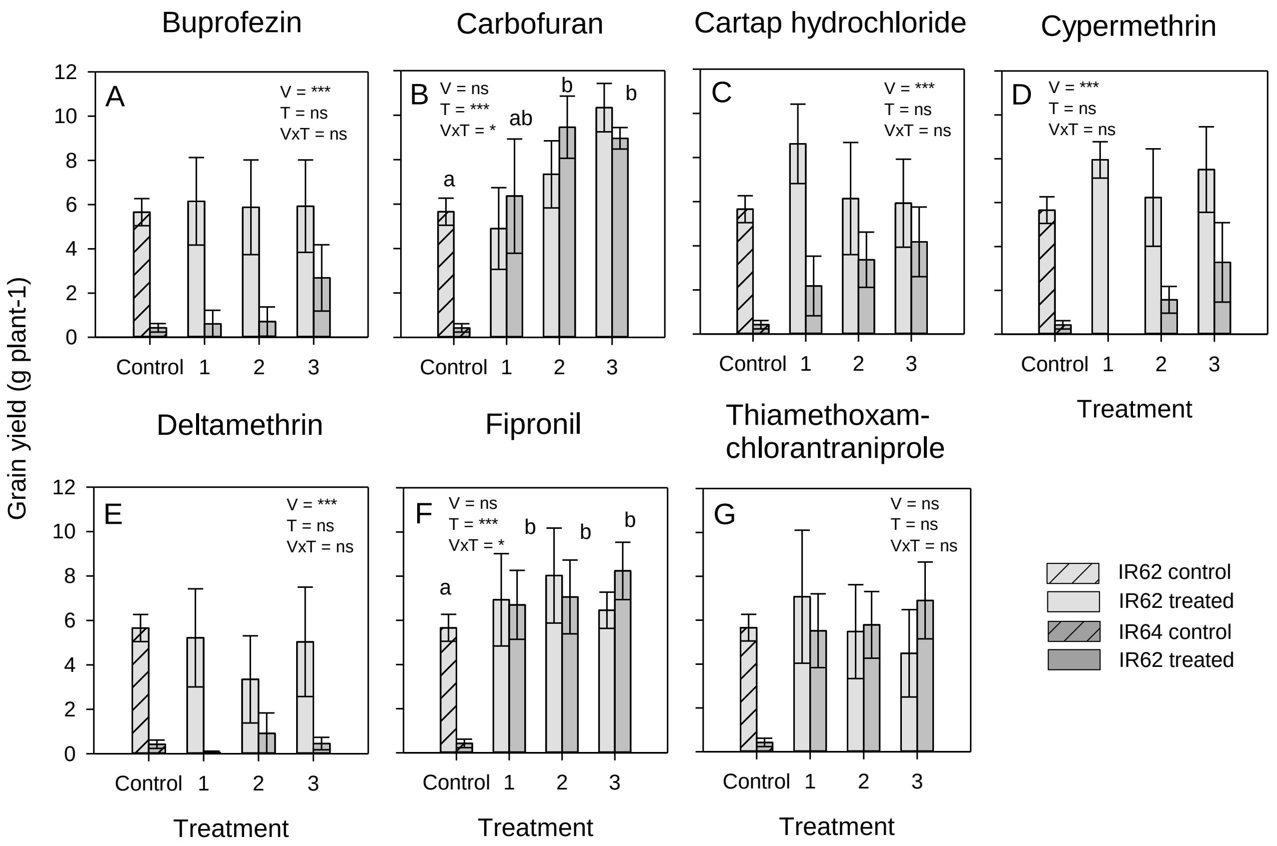

Figure S2: Grain production (yields) for IR62 (resistant) and IR64 (susceptible) rice plants with 0 (control), 1, 2 or 3 applications of A) buprofezin, B) carbofuran, C) cartap hydrochloride, D) cypermethrin, E) deltamethrin, F) fipronil, or G) thiamethoxam + chlorantraniprole. Graphs are redrawn based on data presented in Table 2 of the main text to highlight control plants versus plants treated with each insecticide. Results of univariate GLMs for each insecticide are indicated as V (variety effect), T (treatment effect, including controls), and VxT (variety x treatment interaction). ns = P > 0.05, * = P ≤ 0.05, *** = P ≤ 0.005. Lowercase letters indicate homogenous treatment groups. Standard errors are indicated. For further details of analyses, see Table S2

Table S3: Growth parameters for IR62 (resistant) and IR64 (susceptible) rice varieties infested with brown planthopper and treated with one of seven insecticides in a pot experiment. Plants were treated with one, two or three applications of each insecticide. Numbers are means ± SEM. For further details concerning infested plants, see Table 2, Figure S2, and Table S2; for further details concerning non-infested plants see Table S4.

Variety and Insecticide	Number of Applications	Time to Harvest (days)[1]	Number of Tillers[1]	Plant Height (cm)[1]	Root Length (cm)[1]	Number of Panicles[1]	Above Ground Biomass (g Dry Weight)[1]	Root Biomass (g Dry Weight)[1]
IR62								
Buprofezin	1	79.40±5.60 [ab A]	7.20±1.11	96.72±0.52 [a A]	22.60±2.38 [a]	5.40±1.63 [ab]	8.03±1.69 [a]	3.05±1.08 [abB]
	2	79.80±5.45 [AB]	6.00±2.00	99.94±5.49 [B]	25.50±2.82	5.00±1.48	7.82±2.77	3.56±1.07 [AB]
	3	83.80±2.15 [C]	6.40±3.40	99.60±4.62 [B]	26.84±4.16	5.20±0.58	7.91±3.81	2.32±0.47 [A]
Carbofuran	1	79.60±3.87 [cd]	5.80±1.73	98.18±4.98 [b]	29.80±1.24 [bc]	4.60±1.29 [b]	8.28±1.34 [c]	4.64±1.38 [c]
	2	91.60±2.34	7.60±2.29	101.00±0.89	27.50±1.95	7.00±1.41	10.49±2.70	3.21±0.49
	3	87.80±1.20	6.80±3.24	108.40±5.72	26.40±3.26	6.00±0.89	9.37±3.21	3.56±0.66
Cartap hydrochloride	1	89.00±1.30 [bc]	5.60±1.03	94.70±3.22 [a]	33.20±4.33 [bc]	5.20±0.86 [ab]	8.33±1.03 [ab]	2.81±0.46 [a]
	2	78.40±5.56	7.40±2.24	103.96±3.91	27.20±2.80	4.20±1.74	7.58±2.10	2.00±0.36
	3	82.20±4.69	5.60±3.03	93.28±5.32	21.98±3.93	4.80±1.28	6.90±3.67	2.45±0.69
Cypermethrin	1	87.00±1.10 [b]	7.40±1.51	95.90±2.48 [ab]	29.60±2.89 [abc]	7.00±0.32 [ab]	9.62±1.62 [abc]	3.63±0.80 [abc]
	2	81.00±4.98	6.20±2.58	105.76±5.48	28.20±2.59	4.40±1.21	7.93±2.48	3.77±0.79
	3	84.60±5.15	6.80±3.80	103.18±6.11	34.22±2.98	4.80±1.24	8.90±3.49	1.87±0.46
Deltamethrin	1	77.60±6.65 [a]	7.20±1.58	94.76±2.61 [a]	28.76±1.69 [ab]	4.40±1.83 [a]	8.31±1.47 [a]	2.33±0.59 [abc]
	2	73.80±5.09	7.00±2.63	95.32±3.27	19.70±4.03	3.00±1.38	7.16±2.41	3.87±1.14
	3	77.60±7.19	6.00±3.48	96.10±3.02	21.82±3.28	4.40±1.91	7.00±3.14	1.59±0.75
Fipronil	1	88.20±3.64 [d]	6.80±1.66	93.06±1.89 [a]	32.50±1.43 [c]	5.60±1.44 [b]	9.33±1.26 [bc]	3.78±1.30 [abc]
	2	85.00±3.82	6.40±2.75	97.50±3.16	29.40±4.03	5.20±1.39	8.21±2.88	3.10±0.62
	3	91.80±3.85	6.60±3.40	94.00±3.56	23.80±2.20	6.40±0.51	9.09±3.54	1.89±0.65
Thiamethoxam + chlorantraniliprole	1	80.80±3.32 [bc]	7.00±1.77	101.04±4.39 [ab]	26.66±2.50 [abc]	4.40±1.81 [ab]	9.91±1.34 [bc]	3.62±0.96 [bc]
	2	80.20±3.84	6.00±2.45	96.90±3.94	25.40±2.54	4.00±1.14	6.95±2.77	2.27±0.61
	3	82.00±2.30	5.80±3.20	99.24±4.88	23.10±1.91	3.60±1.47	7.56±3.61	2.05±0.30
Control		85.50±3.41	7.40±0.70	100.16±2.68	28.80±3.33	6.00±0.71	8.80±0.83	3.39±1.02
IR64								
Buprofezin	1	69.80±2.91	4.40±1.51	86.80±3.92	21.40±4.78	1.20±1.20	4.62±1.23	0.94±0.32
	2	74.00±5.24	5.00±2.71	86.46±4.48	23.04±5.25	3.00±1.22	5.61±2.70	1.18±0.46
	3	80.00±4.83	4.80±3.58	89.76±2.76	25.24±4.87	3.00±1.34	6.65±3.88	1.68±0.44

Carbofuran	1	90.00±2.35	5.20±1.80	95.80±4.08	32.40±4.01	4.40±0.75	9.45±1.44	2.50±0.72
	2	90.00±1.10	6.20±2.02	101.30±2.79	33.50±4.00	6.00±0.84	9.51±2.26	2.55±0.61
	3	94.00±2.45	6.20±3.66	104.00±2.23	34.80±4.57	5.40±0.40	11.92±3.27	3.47±0.97
Cartap hydrochloride	1	78.60±4.43	4.60±1.87	90.94±5.23	32.72±4.85	3.00±1.34	6.48±1.55	1.15±0.27
	2	83.40±2.50	5.80±2.73	96.30±1.74	35.90±2.64	4.00±1.10	7.92±2.90	1.99±0.57
	3	86.40±4.13	5.00±3.84	91.64±4.63	30.12±2.93	4.00±1.18	8.20±3.44	1.89±0.36
Cypermethrin	1	67.40±2.06	6.20±1.28	86.38±4.66	24.06±3.89	0.00±0.00	5.98±1.80	1.92±0.73
	2	80.40±1.57	5.80±2.66	100.14±3.35	30.50±3.28	3.40±0.98	7.90±2.86	2.53±1.19
	3	86.60±1.25	5.40±3.87	94.50±3.59	32.80±4.79	4.00±0.71	8.37±3.13	1.98±0.70
Deltamethrin	1	68.40±2.29	5.60±1.12	93.16±2.35	29.86±4.93	0.60±0.60	6.29±1.98	1.80±0.72
	2	72.80±4.13	5.60±2.51	91.90±2.18	30.40±3.06	1.00±1.00	6.20±2.12	2.25±0.74
	3	72.80±3.51	6.00±3.14	92.38±2.08	30.42±2.15	3.40±1.47	7.04±3.48	1.84±0.39
Fipronil	1	89.20±1.36	5.80±1.49	94.80±2.80	40.00±6.75	4.80±0.73	9.90±1.55	2.47±0.13
	2	89.20±1.36	5.80±2.97	94.40±2.29	37.40±3.63	5.20±0.80	8.89±2.40	1.97±0.64
	3	96.40±2.64	6.40±3.03	95.60±3.96	33.70±3.03	6.20±1.07	11.88±3.30	2.16±0.41
Thiamethoxam + chlorantraniliprole	1	84.00±2.07	6.80±1.97	94.94±1.99	36.90±0.78	5.40±1.36	10.60±1.98	5.45±1.78
	2	87.80±1.24	5.60±2.93	96.80±4.42	35.20±2.22	4.80±0.58	9.02±2.14	2.46±0.68
	3	83.40±2.94	7.20±3.53	100.66±2.91	31.40±4.26	5.80±1.02	10.91±3.76	3.53±0.94
Control		70.10±1.81	4.70±0.78	80.60±3.63	24.37±3.00	2.30±1.20	4.43±0.48	1.72±0.69
F-variety (V)		0.512ns	13.213***	13.526***	13.904***	10.141***	0.013ns	7.476**
F-treatment (T)		14.927***	1.072ns	3.645***	3.089**	3.829***	4.878***	2.634*
F-applications (A)		5.563***	0.254ns	3.993*	0.855ns	1.347ns	0.97ns	2.124*
F-V×T		3.432***	1.151ns	2.003ns	2.250*	5.622***	8.035***	9.515***
F-V×A		3.377*	0.643ns	0.078ns	1.141ns	6.931***	5.979***	5.776***
F-control (C)		3.240ns	0.015ns	4.880*	0.816ns	0.055ns	2.780ns	0.005ns
F-C×V		7.779**	2.717ns	7.972***	15.077***	14.634***	5.036*	9.855***

[1]: ns = P > 0.05, * = P ≤ 0.05, ** = P ≤ 0.01, *** = P ≤ 0.005; lowercase letters indicate homogenous treatment (insecticide) groups for IR62 and IR64 based on Tukey pairwise comparisons (P ≤ 0.05); Numerator degrees of freedom for general linear models using the Addelman (1974) method are as follows: variety, 1; treatment, 6; applications, 2; V×T, 5; V×A, 12; control, 1; C×V, 1; denominator degrees of freedom were 164. Non-significant interactions and block effects are not presented.

Table S4: Growth parameters for IR62 (resistant) and IR64 (susceptible) rice varieties treated with one of seven insecticides in a pot experiment. Plants were treated with one, two or three applications of each insecticide. Numbers are means ± SEM. For further details concerning infested plants, see Table 2 and Table S3, respectively

Variety and Insecticide	Number of Applications	Time to Harvest (days)[1]	Number of Tillers[1]	Plant Height (cm)[1]	Root Length (cm)[1]	Number of Panicles[1]	Above Ground Biomass (g Dry Weight)[1]	Root Biomass (g Dry Weight)[1]	Weight of Filled Grains (g Dry Weight)[1]	Number of Filled Grain[1]	Proportion of Grain Unfilled[1]	1000 Grain Weight[1]
IR62												
Buprofezin	1	92.40±1.50	6.80±1.49	99.70±1.53	34.20±1.83	6.80±1.80	10.09±1.03	3.18±0.84	11.55±0.95	604.80±55.08	0.12±0.02	19.18±0.44
	2	90.40±2.06	7.20±2.58	104.60±4.07	27.60±3.22	6.60±2.60	10.89±0.88	3.36±0.69	10.41±0.56	540.80±22.55	0.17±0.02	19.21±0.30
	3	91.80±1.50	6.60±3.60	102.80±3.87	29.90±1.17	6.20±3.20	9.65±0.58	2.48±0.45	9.82±0.94	512.00±42.14	0.11±0.02	19.14±0.58
Carbofuran	1	93.40±0.81	6.00±1.45	97.50±3.15	29.20±0.97	6.00±1.00	9.48±0.87	2.27±0.43	10.12±0.84	509.00±36.96	0.21±0.04	19.82±0.43
	2	91.00±1.84	8.40±2.68	101.40±3.08	27.00±3.13	7.60±2.60	11.30±0.84	4.10±1.49	10.88±0.74	560.40±36.09	0.20±0.04	19.41±0.20
	3	91.80±2.03	6.60±3.40	98.00±1.30	33.30±2.55	6.20±3.20	10.36±0.71	2.35±0.21	10.00±0.50	529.60±21.47	0.13±0.02	18.89±0.50
Cartap hydrochloride	1	92.60±1.69	6.20±1.73	98.00±2.28	33.00±3.52	6.00±1.00	10.30±0.81	1.94±0.32	10.32±0.84	503.00±45.03	0.13±0.03	20.66±1.04
	2	90.80±1.59	6.20±2.80	102.30±3.33	28.60±1.91	5.80±2.80	9.81±0.79	3.30±0.72	10.18±0.83	541.60±21.92	0.10±0.02	18.67±0.80
	3	90.80±2.01	5.40±3.51	103.40±3.03	29.20±2.40	5.40±3.40	9.40±0.31	3.59±0.92	9.34±0.40	475.20±24.52	0.15±0.03	19.69±0.34
Cypermethrin	1	91.40±2.04	7.00±1.63	100.10±3.50	31.80±1.39	6.40±1.40	10.45±0.39	2.23±0.37	9.83±0.71	532.20±59.76	0.18±0.03	18.79±0.78
	2	94.00±3.52	5.60±2.40	101.60±4.88	24.90±4.76	5.00±2.00	8.55±0.91	1.57±0.45	7.56±1.30	419.60±70.38	0.21±0.10	17.84±0.60
	3	92.20±2.20	6.80±3.73	98.00±2.59	28.60±3.50	6.60±3.60	10.60±1.10	2.53±0.32	9.23±0.74	481.20±37.09	0.22±0.04	19.17±0.14
Deltamethrin	1	92.40±1.50	6.20±1.80	100.20±4.40	35.00±2.65	5.80±1.80	9.92±0.87	3.11±0.65	9.93±0.67	502.20±39.21	0.15±0.03	19.87±0.57
	2	90.60±2.75	6.20±2.86	103.00±2.95	28.60±3.63	6.20±2.20	9.41±0.51	3.00±0.41	9.83±0.82	509.60±38.89	0.14±0.03	19.27±0.50
	3	91.00±1.84	5.80±3.58	103.20±4.14	28.40±2.64	5.80±3.80	9.02±0.72	1.64±0.36	9.82±0.78	500.80±29.06	0.17±0.03	19.51±0.54
Fipronil	1	94.00±0.84	6.80±1.66	97.60±3.78	26.60±4.32	6.60±1.60	9.48±0.70	1.81±0.29	10.94±1.39	572.60±57.50	0.17±0.03	18.89±0.76
	2	90.60±2.75	8.40±2.33	100.60±6.35	32.00±3.89	8.20±2.20	10.71±1.86	2.05±0.30	11.10±1.85	573.40±106.14	0.25±0.03	19.60±0.35
	3	93.40±0.81	6.60±3.51	98.20±3.14	33.60±2.58	6.40±3.40	9.69±0.57	2.18±0.33	10.03±0.43	522.60±13.20	0.14±0.02	19.19±0.54
Thiamethoxam + chlorantraniliprole	1	89.00±2.53	7.20±1.49	98.30±2.88	26.20±2.89	6.40±1.40	10.26±0.55	3.76±1.34	10.41±0.88	558.40±58.08	0.18±0.05	18.83±0.69
	2	92.80±1.53	7.20±2.20	101.40±3.12	33.20±3.18	6.80±2.80	11.07±0.47	2.69±0.29	10.82±0.48	580.60±27.51	0.12±0.02	18.66±0.20
	3	90.80±2.33	8.00±3.55	99.00±2.89	29.40±1.69	7.20±3.20	10.26±0.49	3.49±0.35	9.29±0.58	519.20±18.99	0.19±0.06	17.86±0.75
Control		94.20±0.72	6.90±0.64	104.45±4.30	29.45±1.61	6.50±0.50	9.62±1.12	2.39±0.37	10.02±0.69	531.60±51.72	0.15±0.03	19.06±0.61
IR64												

Buprofezin	1	94.80±0.73	5.40±1.51	104.80±5.48	40.40±3.54	5.40±1.40	10.90±1.34	1.70±0.20	9.62±0.57	455.40±21.05	0.14±0.05	21.14±0.80
	2	95.20±0.73	6.40±2.51	100.40±4.01	39.40±2.06	5.60±2.60	11.93±1.49	2.05±0.67	8.80±0.77	400.40±33.63	0.12±0.02	21.96±0.31
	3	95.40±1.40	5.00±3.32	100.20±1.46	43.40±1.17	4.80±3.80	11.20±0.75	2.38±0.37	8.84±0.47	405.60±20.29	0.10±0.01	21.79±0.18
Carbofuran	1	96.80±0.20	6.00±1.55	100.80±4.35	41.80±3.40	5.60±1.60	13.86±1.45	2.27±0.25	9.37±0.94	441.80±47.96	0.23±0.03	21.25±0.42
	2	94.60±0.60	5.80±2.73	101.90±4.20	41.00±2.21	5.20±2.20	12.33±0.98	2.09±0.25	9.75±0.51	439.60±23.03	0.12±0.02	22.18±0.24
	3	92.20±2.11	7.40±3.78	104.60±3.82	38.60±2.46	7.00±3.00	12.57±1.29	3.66±1.60	9.80±1.14	482.80±69.72	0.20±0.05	20.68±1.02
Cartap hydrochloride	1	95.00±0.84	6.00±1.63	99.70±1.93	40.60±1.66	5.60±1.60	12.69±1.38	2.29±0.44	9.62±1.54	442.60±65.27	0.16±0.03	21.52±0.79
	2	95.60±0.68	7.60±2.60	104.00±3.58	41.40±4.30	7.60±2.60	13.96±1.93	2.59±0.49	9.51±0.99	457.40±47.82	0.22±0.09	20.85±0.86
	3	94.00±1.05	6.40±3.03	101.10±4.38	33.20±3.62	5.20±3.20	10.22±0.94	2.46±0.54	8.62±0.82	428.80±49.73	0.17±0.04	20.31±0.58
Cypermethrin	1	93.00±2.32	5.80±1.86	99.20±2.58	31.20±5.53	5.40±1.40	11.10±1.70	1.85±0.42	8.18±1.33	396.40±61.94	0.18±0.05	20.68±1.03
	2	92.60±1.40	6.40±2.93	99.80±3.30	36.60±2.73	6.00±2.00	13.24±1.73	3.11±0.50	9.09±0.87	416.80±34.29	0.15±0.02	21.70±0.62
	3	93.40±1.86	5.40±3.51	102.30±3.17	39.00±3.21	5.20±3.20	11.94±0.84	2.26±0.16	8.60±0.27	432.00±21.07	0.17±0.03	20.04±0.91
Deltamethrin	1	95.80±0.97	5.00±1.71	101.00±4.84	38.40±1.40	5.00±1.00	12.25±1.39	2.01±0.57	8.89±1.21	416.80±60.43	0.19±0.04	21.62±1.04
	2	93.40±1.78	6.20±2.66	100.00±2.07	40.80±5.40	5.80±2.80	11.61±1.38	2.15±0.24	9.63±0.77	456.00±46.22	0.13±0.01	21.30±0.81
	3	93.20±1.77	5.60±3.93	103.50±4.24	42.00±4.11	5.00±3.00	12.33±0.84	2.22±0.33	9.64±0.59	437.20±25.56	0.11±0.02	22.04±0.35
Fipronil	1	95.40±1.21	6.20±1.73	98.10±6.23	39.00±1.70	5.80±1.80	12.04±0.94	2.25±0.26	9.95±1.21	452.00±55.45	0.13±0.03	22.03±0.15
	2	94.20±0.80	6.20±2.73	96.70±3.06	37.60±2.36	5.60±2.60	11.35±1.08	2.95±0.62	8.96±0.67	418.60±25.90	0.19±0.02	21.37±0.63
	3	94.40±0.68	6.60±3.60	99.70±3.81	35.00±2.97	5.60±3.60	13.14±1.45	2.72±1.03	9.87±0.64	452.60±38.70	0.20±0.02	22.01±0.87
Thiamethoxam + chlorantraniliprole	1	94.80±0.73	5.80±1.58	102.20±3.62	40.00±1.00	5.60±1.60	12.98±1.18	2.39±0.41	9.54±0.31	441.80±30.48	0.17±0.03	21.83±0.96
	2	93.00±0.55	6.00±2.63	96.40±1.60	36.60±3.49	5.60±2.60	10.90±1.23	2.74±0.67	9.14±0.95	416.60±47.27	0.14±0.02	22.07±0.81
	3	96.40±2.40	7.60±3.51	96.00±1.76	33.80±4.16	7.00±3.00	14.69±1.10	2.78±0.48	8.78±1.16	403.60±46.57	0.22±0.07	21.54±0.53
Control		94.80±0.70	6.70±0.51	96.40±2.01	39.85±2.32	6.40±0.40	10.78±0.71	1.91±0.31	10.14±0.54	456.50±24.71	0.16±0.01	22.23±0.20
F-variety		18.781***	6.808***	0.026ns	64.805***	9.784***	43.775***	1.761ns	7.231***	35.328***	0.099ns	98.728***
F-treatment		0.287ns	1.823ns	1.064ns	0.936ns	1.209ns	0.748ns	1.002ns	1.235ns	0.729ns	1.488ns	0.972ns
F-applications		1.128ns	1.823ns	0.496ns	0.252ns	1.048ns	0.062ns	1.008ns	0.803ns	0.425ns	0.049ns	0.587ns
F-control		1.414ns	0.487ns	0.001ns	0.021ns	0.634ns	1.444ns	0.741ns	0.347ns	0.155ns	0.099ns	0.436ns

[1]: ns = P > 0.05, *** = P ≤ 0.005; Numerator degrees of freedom for general linear models using the Addelman (1974) method are as follows: variety, 1; treatment, 6; applications, 2; control, 1; denominator degrees of freedom are 164. Non-significant interactions and block effects are not presented.

Table S5: Planthopper density (number per g plant) on (A) IR62 (resistant) and (B) IR64 (susceptible) rice plants treated at 20 or 50 days after sowing with one of seven insecticides, and on non-treated controls. The numbers of plants surviving are also indicated. All plants were infested with four gravid female BPH at 40 DAS and again with two gravid females at 60 DAS. Numbers are means ± SEM

Variety and Insecticide	Time of Application	Plant Survival (Proportion) [1]	Number of BPH per g of Plant[1]
IR62			
Buprofezin	20	0.60±0.24 [bc]	122.44±56.41
	50	1.00±0.00	18.60±7.82
Carbofuran	20	0.40±0.24 [c]	454.61±283.76
	50	0.80±0.20	6.56±5.39
Cartap hydrochloride	20	1.00±0.00 [bc]	59.07±51.49
	50	1.00±0.00	15.46±7.16
Cypermethrin	20	1.00±0.00 [bc]	101.22±37.06
	50	0.60±0.24	44.42±33.13
Deltamethrin	20	0.60±0.24 [b]	171.56±27.65
	50	0.60±0.24	152.24±84.96
Fipronil	20	0.60±0.24 [bc]	201.88±183.95
	50	0.60±0.24	68.54±46.70
Thiamethoxam + chlorantraniliprole	20	0.60±0.24 [bc]	96.24±60.93
	50	0.60±0.24	114.61±76.54
Control		0.40±0.24	226.35±55.00
IR64			
Buprofezin	20	0.00±0.00	265.09±57.64
	50	0.20±0.20	396.99±122.81
Carbofuran	20	0.80±0.20	65.86±62.27
	50	1.00±0.00	0.07±0.07
Cartap hydrochloride	20	0.00±0.00	312.40±57.53
	50	0.00±0.00	403.00±130.55
Cypermethrin	20	0.00±0.00	196.33±72.65
	50	0.40±0.24	298.60±145.28
Deltamethrin	20	0.00±0.00	219.46±49.36
	50	0.00±0.00	164.19±29.58
Fipronil	20	1.00±0.00	96.50±76.69
	50	0.40±0.24	134.15±48.67
Thiamethoxam + chlorantraniliprole	20	0.60±0.24	200.98±14.36
	50	0.20±0.20	383.13±281.94
Control		0.00±0.00	279.67±56.52
F-variety (V)		23.222***	7.746**
F-treatment (T)		2.501*	0.663ns
F-day		0.031ns	0.501ns
F-V×T		5.692***	4.180***
F-control		4.194*	1.365ns

[1]: ns = P > 0.05, * = P ≤ 0.05, ** = P ≤ 0.01, *** = P ≤ 0.005; lowercase letters indicate homogenous treatment (insecticide) groups for IR62 and IR64 based on Tukey pairwise comparisons (P ≤ 0.05); Numerator degrees of freedom for general linear models using the Addelman (1974) method are as follows: variety, 1; treatment, 6; day, 1; V×T, 5; control, 1; denominator degrees of freedom are 164. Non-significant interactions and block effects are not presented.

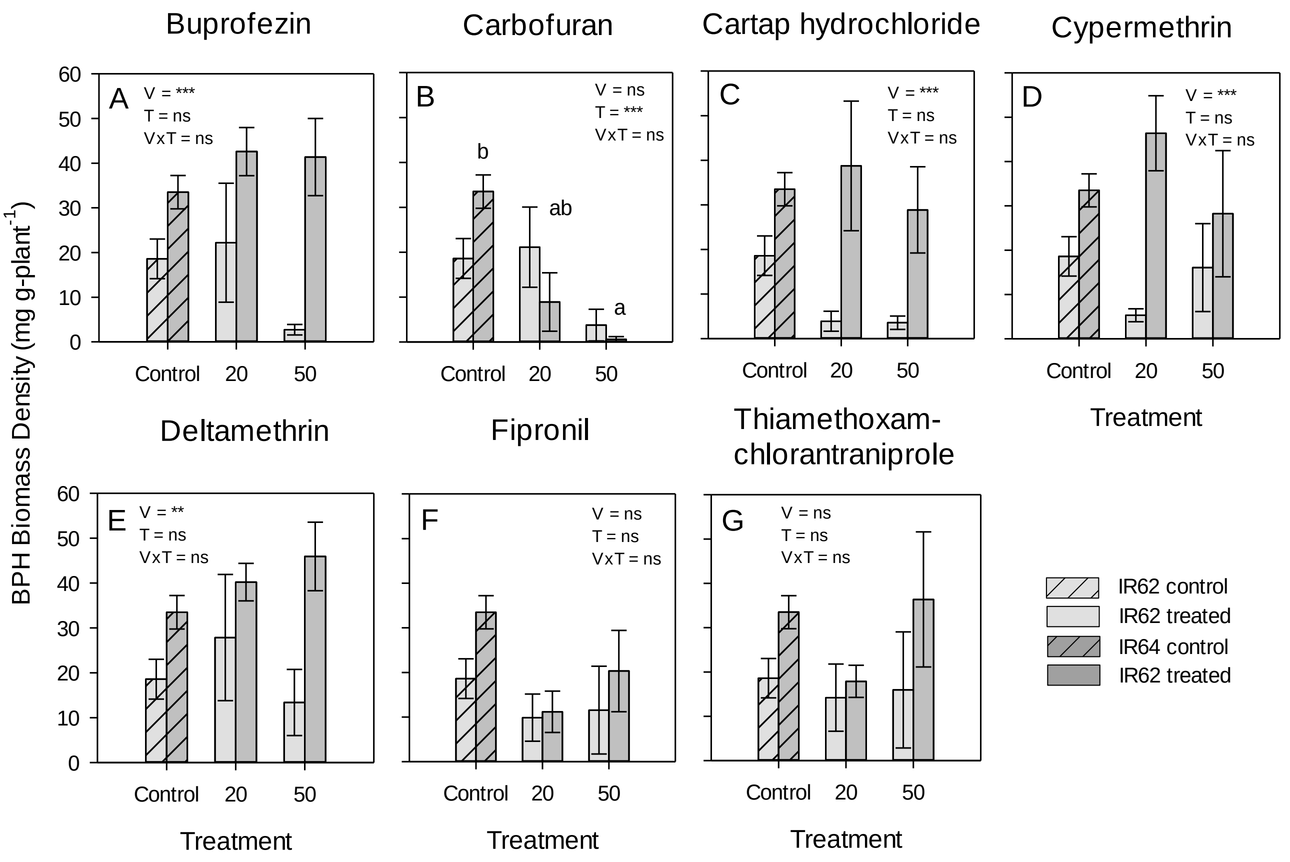

Figure S3: Biomass density of brown planthopper (BPH) on IR62 (resistant) and IR64 (susceptible) rice plants with 0 applications (control), and with applications at 20 and 50 DAS of A) buprofezin, B) carbofuran, C) cartap hydrochloride, D) cypermethrin, E) deltamethrin, F) fipronil, or G) thiamethoxam + chlorantraniprole. Graphs are redrawn based on data presented in Figure 1 of the main text to highlight control plants versus plants treated with each insecticide. Results of univariate GLMs for each insecticide are indicated as V (variety effect), T (treatment effect, including controls), and VxT (variety x treatment interaction). ns = $P > 0.05$, ** = $P \leq 0.01$, *** = $P \leq 0.005$. Lowercase letters indicate homogenous treatment groups. Standard errors are indicated. For further details of analyses, see Table S6.

Table S6: Results from univariate GLMs for planthopper biomass density (biomass per g plant), and density (number per g plant). Results from analyses of plant survival and plant yields (weight of grain) are also presented. GLMs were conducted separately for each insecticide product. Results from comparative analyses of all seven insecticides are presented in the main text (Figure 3, Table 3)

**Parameters**[1]	**Sources of Variation**[2]	**F-values**[3]						
		**Buprofezin**	**Carbofuran**	**Cartap hydrochloride**	**Cypermethrin**	**Deltamethrin**	**Fipronil**	**Thiamethoxam + chlorantraniprole**
Biomass density (mg per g plant)	Variety (V)	20.791***	0.001ns	16.971***	10.750***	8.876**	2.218ns	2.614ns
	Treatment (T)	1.244ns	9.189***	0.874ns	0.135ns	0.476ns	2.649ns	0.696ns
	VxT	7.761ns	3.071ns	0.899ns	1.768ns	0.901ns	0.493ns	0.376ns
Density (number per g plant)	Variety (V)	10.868***	1.287ns	17.894***	4.329*	0.623ns	0.004ns	1.766ns
	Treatment (T)	0.379ns	2.827ns	0.520ns	0.963ns	1.331ns	1.550ns	0.407ns
	VxT	2.787ns	1.900ns	3.151ns	0.900ns	0.074ns	0.586ns	0.368ns
Plant survival (proportion)	Variety (V)	19.756***	0.192ns	96.000***	12.549***	13.913***	0.192ns	2.254ns
	Treatment (T)	3.171ns	7.115***	6.000**	1.765ns	0.217ns	5.192**	1.690ns
	VxT	0.732ns	2.500ns	6.000**	2.549ns	0.217ns	2.500ns	0.563ns
Yield (g per plant) - infested	Variety (V)	49.461***	0.799ns	34.694***	14.426***	22.893***	3.296ns	3.658ns
	Treatment (T)	3.109ns	4.129*	3.501*	1.009ns	0.096ns	4.560*	2.233ns
	VxT	1.488ns	1.222ns	0.599ns	2.874ns	0.270ns	0.868ns	0.230ns

1: Means for density and plant survival are presented in Table S5; means for biomass density are presented in Figure 3 and Figure S3; means for yield are presented in Table 3 and Figure S4

2: Degrees of freedom for variety = 1,20; treatment = 3,20; and V×T interaction = 3,20. Block effects are not presented

3: ns = $P > 0.05$; * = $P \leq 0.05$; ** = $P \leq 0.01$; *** = $P \leq 0.05$

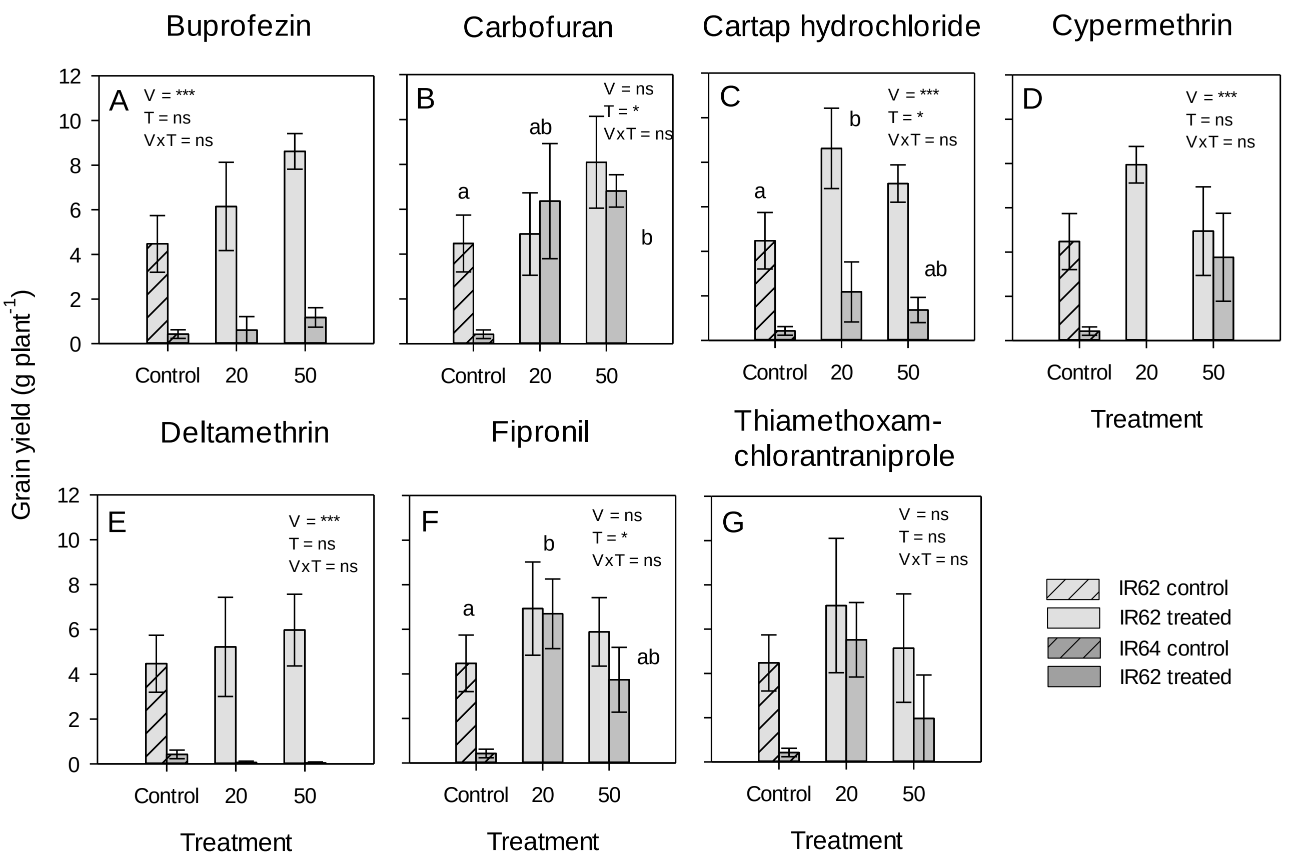

Figure S4: Grain production (yields) for IR62 (resistant) and IR64 (susceptible) rice plants with 0 applications (control), and with applications at 20 and 50 DAS of A) buprofezin, B) carbofuran, C) cartap hydrochloride, D) cypermethrin, E) deltamethrin, F) fipronil, or G) thiamethoxam + chlorantraniprole. Graphs are redrawn based on data presented in Table 3 of the main text to highlight control plants versus plants treated with each insecticide. Results of univariate GLMs for each insecticide are indicated as V (variety effect), T (treatment effect, including controls), and VxT (variety x treatment interaction). Ns = P > 0.05, * = P ≤ 0.05, *** = P ≤ 0.005. Lowercase letters indicate homogenous treatment groups. Standard errors are indicated. For further details of analyses, see Table S6.

Table S7: Growth parameters for IR62 (resistant) and IR64 (susceptible) rice varieties infested with brown planthopper and treated with one of seven insecticides in a pot experiment. Rice plants were treated at 20 or 50 days after sowing. Numbers are means ± SEM. For further details concerning infested and non-infested plants see Table 3 and Table S8, respectively

Variety and Insecticide	Application Time (days)	Time to Harvest (days) [1]	Number of Tillers[1]	Plant Height (cm) [1]	Root Length (cm) [1]	Number of Panicles[1]	Above Ground Biomass (g Dry Weight) [1]	Root Biomass (g Dry Weight) [1]
IR62								
Buprofezin	20	79.40±5.60 [abc]	7.20±1.11	96.72±0.52	22.60±2.38	5.40±1.63	8.03±1.69	3.05±1.08
	50	91.00±3.86	7.20±1.20	96.60±1.98	31.20±2.78	6.20±0.58	8.93±1.12	3.76±1.00
Carbofuran	20	79.60±3.87 [c]	5.80±0.73	98.18±4.98	29.80±1.24	4.60±1.29	8.28±1.34	4.64±1.38
	50	81.40±5.68	5.60±1.29	88.02±12.74	24.44±5.57	4.80±1.20	7.22±1.81	3.72±1.35
Cartap hydrochloride	20	89.00±1.30 [bc]	5.60±1.03	94.70±3.22	33.20±4.33	5.20±0.86	8.33±1.03	2.81±0.46
	50	90.40±2.01	7.40±0.68	97.10±3.78	30.40±2.79	6.60±0.68	9.03±0.58	2.17±0.24
Cypermethrin	20	87.00±1.10 [abc]	7.40±0.51	95.90±2.48	29.60±2.89	7.00±0.32	9.62±0.62	3.63±0.80
	50	81.40±6.71	6.60±0.68	98.34±5.82	29.32±0.76	4.00±1.48	7.08±1.39	2.07±0.42
Deltamethrin	20	77.60±6.65 [a]	7.20±0.58	94.76±2.61	28.76±1.69	4.40±1.83	8.31±1.47	2.33±0.59
	50	79.60±5.52	6.00±0.55	97.06±7.11	22.40±3.88	4.40±1.21	7.70±0.69	2.08±0.43
Fipronil	20	88.20±3.64 [c]	6.80±0.66	93.06±1.89	32.50±1.43	5.60±1.44	9.33±1.26	3.78±1.30
	50	80.00±4.32	7.20±1.02	95.22±4.36	23.60±3.58	4.60±1.21	7.47±0.75	2.67±0.46
Thiamethoxam + chlorantraniliprole	20	80.80±3.32 [abc]	7.00±0.77	101.04±4.39	26.66±2.50	4.40±1.81	9.91±1.34	3.62±0.96
	50	88.40±1.12	6.00±1.58	77.60±8.27	35.72±12.73	4.00±1.64	5.55±1.93	2.24±1.42
Control		80.00±5.30	7.80±0.51	96.47±4.37	28.39±3.21	4.80±1.36	8.44±1.00	3.71±0.90
IR64								
Buprofezin	20	69.80±2.91	4.40±0.51	86.80±3.92	21.40±4.78	1.20±1.20	4.62±1.23	0.94±0.32
	50	86.20±3.35	4.60±0.93	83.28±4.15	29.82±5.19	3.60±1.21	5.25±1.93	3.20±2.29
Carbofuran	20	90.00±2.35	5.20±0.80	95.80±4.08	32.40±4.01	4.40±0.75	9.45±1.44	2.50±0.72

	50	95.00±1.48	5.60±0.81	90.90±4.23	37.60±1.50	5.40±0.75	10.25±0.29	2.31±0.39
Cartap hydrochloride	20	78.60±4.43	4.60±0.87	90.94±5.23	32.72±4.85	3.00±1.34	6.48±1.55	1.15±0.27
	50	76.40±2.23	6.80±1.53	92.48±4.71	25.70±3.34	5.00±1.30	8.05±2.13	2.04±0.70
Cypermethrin	20	67.40±2.06	6.20±1.28	86.38±4.66	24.06±3.89	0.00±0.00	5.98±1.80	1.92±0.73
	50	84.60±4.87	5.00±0.71	86.44±5.37	29.20±3.83	3.40±0.81	5.78±1.58	1.27±0.31
Deltamethrin	20	68.40±2.29	5.60±1.12	93.16±2.35	29.86±4.93	0.60±0.60	6.29±1.98	1.80±0.72
	50	69.20±1.39	5.20±0.58	86.04±1.34	32.30±4.76	0.40±0.40	4.93±1.18	1.65±0.50
Fipronil	20	89.20±1.36	5.80±0.49	94.80±2.80	40.00±6.75	4.80±0.73	9.90±0.55	2.47±0.13
	50	84.60±5.46	4.60±1.21	83.90±3.36	30.28±5.78	3.80±1.53	4.81±1.03	1.29±0.39
Thiamethoxam + chlorantraniliprole	20	84.00±2.07	6.80±0.97	94.94±1.99	36.90±0.78	5.40±1.36	10.60±0.98	5.45±1.78
	50	73.80±6.92	4.00±0.32	80.38±12.21	22.84±6.78	1.00±1.00	4.10±1.41	0.98±0.36
Control		70.10±1.81	4.70±0.78	80.60±3.63	24.37±3.00	2.30±1.20	4.43±1.48	1.72±0.69
F-variety (V)		10.528***	20.256***	13.082***	0.132ns	20.697***	10.872***	9.923***
F-treatment (T)		4.723***	0.269ns	0.480ns	0.773ns	2.290*	1.208ns	1.266ns
F-day (D)		2.556ns	0.635ns	5.571*	0.443ns	0.037ns	7.029**	3.185ns
F-V×T		5.716***	0.859ns	0.883ns	1.404ns	2.365*	2.195ns	0.686ns
F-T×D		2.712*	1.813ns	1.953ns	1.608ns	1.547ns	3.038**	2.242*
F-control		5.638*	0.168ns	0.759ns	0.902ns	0.335ns	1.208ns	0.059ns

[1]: ns = P > 0.05, * = P ≤ 0.05, *** = P ≤ 0.005; lowercase letters indicate homogenous treatment (insecticide) groups for IR62 and IR64 based on Tukey pairwise comparisons (P ≤ 0.05); Numerator degrees of freedom for general linear models using the Addelman (1974) method are as follows: variety, 1; treatment, 6; day, 1; V×T, 5; T×D, 6; control, 1; denominator degrees of freedom are 122. Non-significant interactions and block effects are not presented.

Table S8: Growth parameters for IR62 (resistant) and IR64 (susceptible) rice varieties treated with one of seven insecticides in a pot experiment. Rice plants were treated at 20 or 50 days after sowing. Numbers are means ± SEM. For further details concerning infested plants see Table 3 and Table S7, respectively

Variety and insecticide	Application Time (days)	Time to Harvest (days)[1]	Number of Tillers[1]	Plant Height (cm)[1]	Root Length (cm)[1]	Number of Panicles[1]	Above Ground Biomass (g Dry Weight)[1]	Root Biomass (g Dry Weight)[1]	Weight of Filled Grain (g Dry Weight)[1,2]	Number of Filled Grain[1]	Proportion of Grain Unfilled[1]	1000 Grain Weight[1]
IR62												
Buprofezin	20	92.40±1.50	6.80±0.49	99.70±1.53	34.20±1.83	6.80±0.49	10.09±1.03	3.18±0.84	11.55±0.95	604.80±55.08	0.12±0.02	19.18±0.44
	50	89.80±2.50	6.60±0.60	101.20±3.54	31.40±2.80	6.00±0.55	10.40±0.87	3.74±0.95	9.22±0.84	505.40±43.90	0.10±0.02	18.28±0.68
Carbofuran	20	93.40±0.81	6.00±0.45	97.50±3.15	29.20±0.97	6.00±0.45	9.48±0.87	2.27±0.43	10.12±0.84	509.00±36.96	0.17±0.06	19.82±0.43
	50	89.00±1.92	6.60±0.93	97.30±5.00	31.00±4.02	6.60±0.93	9.26±0.40	3.06±0.45	9.32±0.65	491.00±26.64	0.10±0.02	19.01±1.11
Cartap hydrochloride	20	92.60±1.69	6.20±0.73	98.00±2.28	33.00±3.52	6.00±0.55	10.30±0.81	1.94±0.32	10.32±0.84	503.00±45.03	0.13±0.03	20.66±1.04
	50	92.80±1.53	6.60±0.60	100.80±4.93	29.80±1.98	6.00±0.55	8.86±0.49	2.64±0.61	8.04±0.60	428.20±31.09	0.18±0.04	18.80±0.57
Cypermethrin	20	91.40±2.04	7.00±0.63	100.10±3.50	31.80±1.39	6.40±0.40	10.45±0.39	2.23±0.37	9.83±0.71	532.20±59.76	0.18±0.03	18.79±0.78
	50	91.00±2.32	6.20±0.37	99.30±4.13	33.20±1.74	6.20±0.37	8.70±0.50	2.36±0.29	8.98±0.76	502.00±40.12	0.14±0.02	17.92±0.64
Deltamethrin	20	92.40±1.50	6.20±0.80	100.20±4.40	35.00±2.65	5.80±0.58	9.92±0.87	3.11±0.65	9.93±0.67	502.20±39.21	0.15±0.03	19.87±0.57
	50	92.75±2.10	5.00±0.41	96.88±3.27	28.75±1.70	4.75±0.63	7.68±0.63	2.42±0.58	7.64±1.44	401.25±75.19	0.11±0.02	19.16±0.84
Fipronil	20	94.00±0.84	6.80±0.66	97.60±3.78	26.60±4.32	6.60±0.68	9.48±0.70	1.81±0.29	10.94±1.39	572.60±57.50	0.17±0.03	18.89±0.76
	50	92.60±1.54	6.20±0.97	95.00±3.90	29.00±1.97	5.80±0.80	8.72±1.06	1.52±0.22	8.83±1.09	447.60±47.08	0.20±0.05	19.55±0.45
Thiamethoxam + chlorantraniliprole	20	89.00±2.53	7.20±0.49	98.30±2.88	26.20±2.89	6.40±0.40	10.26±0.55	3.76±1.34	10.41±0.88	558.40±58.08	0.18±0.05	18.83±0.69
	50	92.00±2.10	6.20±0.86	98.70±4.93	33.60±1.36	5.60±0.51	8.05±0.75	2.13±0.56	8.70±0.70	483.20±40.90	0.12±0.03	18.07±0.65
Control		94.20±0.72	6.90±0.64	104.45±4.30	29.45±1.61	6.50±0.50	9.62±1.12	2.39±0.37	10.02±0.69	531.60±51.72	0.15±0.03	19.04±0.61
IR64												
Buprofezin	20	94.80±0.73	5.40±0.51	104.80±5.48	40.40±3.54	5.40±0.51	10.90±1.34	1.70±0.20	9.62±0.57	455.40±21.05	0.14±0.05	21.14±0.80
	50	93.40±2.71	6.40±0.75	97.20±4.59	40.40±2.68	5.40±0.40	10.18±1.04	2.03±0.31	8.81±0.84	416.00±33.67	0.14±0.02	21.07±0.36
Carbofuran	20	96.80±0.20	6.00±0.55	100.80±4.35	41.80±3.40	5.60±0.51	13.86±1.45	2.27±0.25	9.37±0.94	441.80±47.96	0.23±0.03	21.25±0.42
	50	94.20±1.24	5.80±0.73	99.80±1.35	44.00±2.47	5.60±0.60	11.79±1.07	2.58±0.62	9.85±0.37	438.60±15.89	0.15±0.02	22.45±0.25
Cartap hydrochloride	20	95.00±0.84	6.00±0.63	99.70±1.93	40.60±1.66	5.60±0.51	12.69±1.38	2.29±0.44	9.62±1.54	442.60±65.27	0.16±0.03	21.52±0.79
	50	94.80±0.92	5.60±0.87	97.10±5.52	39.40±4.52	5.60±0.87	10.09±2.20	1.75±0.43	8.16±1.50	377.60±65.10	0.15±0.05	21.34±0.57
Cypermethrin	20	93.00±2.32	5.80±0.86	99.20±2.58	31.20±5.53	5.40±0.68	11.10±1.70	1.85±0.42	8.18±1.33	396.40±61.94	0.18±0.05	20.68±1.03

	50	94.20±0.73	6.00±0.84	101.70±4.32	36.00±1.79	5.60±0.60	12.83±1.79	2.74±0.60	9.07±0.35	419.40±14.61	0.10±0.02	21.63±0.45
Deltamethrin	20	95.80±0.97	5.00±0.71	101.00±4.84	38.40±1.40	5.00±0.71	12.25±1.39	2.01±0.57	8.89±1.21	416.80±60.43	0.19±0.04	21.62±1.04
	50	93.60±0.40	5.20±0.49	99.00±5.06	37.20±1.98	4.80±0.58	10.74±0.88	2.30±0.26	8.80±0.67	414.20±36.61	0.09±0.01	21.36±0.46
Fipronil	20	95.40±1.21	6.20±0.73	98.10±6.23	39.00±1.70	5.80±0.80	12.04±0.94	2.25±0.26	9.95±1.21	452.00±55.45	0.13±0.03	22.03±0.15
	50	95.60±0.68	5.80±0.73	93.40±3.37	38.60±1.66	5.40±0.68	11.25±0.85	2.23±0.35	7.80±0.47	353.80±26.90	0.15±0.01	22.17±0.59
Thiamethoxam + chlorantraniliprole	20	94.80±0.73	5.80±0.58	102.20±3.62	40.00±1.00	5.60±0.51	12.98±1.18	2.39±0.41	9.54±0.31	441.80±30.48	0.17±0.03	21.83±0.96
	50	94.20±1.02	5.80±0.80	100.60±4.48	38.40±3.50	5.20±0.73	10.50±1.53	1.85±0.40	8.74±1.37	403.20±59.54	0.11±0.01	21.51±0.41
Control		94.80±0.70	6.70±0.51	96.40±2.01	39.85±2.32	6.40±0.68	10.78±0.71	1.91±0.31	10.14±0.54	456.50±24.71	0.16±0.01	22.23±0.20
F-variety (V)		26.001***	6.302**	0.461ns	65.149***	0.411ns	31.064***	4.784*	2.389ns	24.418***	0.190ns	101.425***
F-treatment (T)		1.042ns	0.974ns	0.614ns	1.179ns	0.051ns	0.336ns	0.963ns	0.618ns	0.886ns	0.793ns	1.354ns
F-day (D)		1.868ns	0.465ns	0.867ns	0.057ns	0.076ns	8.791***	0.011ns	11.459***	9.947***	7.423**	1.208ns
F-V×T		0.445ns	0.088ns	0.187ns	2.491*	0.008ns	1.036ns	2.146ns	0.312ns	0.621ns	1.066ns	0.786ns
F-V×D		0.002ns	0.833ns	0.512ns	0.018ns	0.026ns	0.001ns	0.175ns	3.054ns	1.597ns	0.761ns	3.781*
F-control		1.342ns	2.116ns	0.209ns	0.024ns	0.130ns	0.193ns	0.352ns	1.358ns	0.974ns	0.190ns	0.497ns

[1]: ns = P > 0.05, * = P ≤ 0.05, ** = P ≤ 0.01, *** = P ≤ 0.005; Numerator degrees of freedom for general linear models using the Addelman (1974) method are as follows: variety, 1; treatment, 6; day, 1; V×T, 5; V×T, 6; control, 1; denominator degrees of freedom are 124. Non-significant interactions and block effects are not presented. [2]: Data related to yields (i.e., grain weights) are also presented in Figure 5 in the main text.
